# Time reference in aphasia: are there differences between tenses and aphasia fluency type? A systematic review and individual participant data meta-analysis

**DOI:** 10.3389/fpsyg.2024.1322539

**Published:** 2024-02-08

**Authors:** Natacha Cordonier, Evodie Schaffner, Lana Zeroual, Marion Fossard

**Affiliations:** Institut des Sciences Logopédiques, Faculté des Lettres et Sciences Humaines, University of Neuchâtel, Neuchâtel, Switzerland

**Keywords:** aphasia, agrammatism, inflection, tense, time reference, verbs

## Abstract

Time reference is used to build the temporal framework of discourse and is essential in ensuring efficient communication. Several studies have reported time reference deficits in fluent and non-fluent aphasia and have shown that tenses (past, present, future) are not all impaired to the same extent. However, there is little consensus on the dissociations between tenses, and the question of the influence of the type of aphasia (fluent vs. non-fluent) on time reference remains open. Therefore, a systematic review and an individual participant data meta-analysis (or mega-analysis) were conducted to determine (1) whether one tense is more impaired than another in fluent and non-fluent aphasia and, if so, (2) which task and speaker-related factors moderate tense effects. The systematic review resulted in 35 studies reporting the performance in time reference of 392 participants. The mega-analysis was then performed on 23 studies for a total of 232 participants and showed an alteration of past tense compared to present and future tenses in both types of aphasia. The analysis also showed a task and an age effect on time reference but no gender effect, independently of tenses. These results add to our knowledge of time reference in aphasia and have implications for future therapies.

## 1 Introduction

Temporality, the time lived by consciousness, is an essential mental construct used to structure our experience. Time reference, the linguistic expression of temporality, is generally used to build the temporal framework of discourse, indicating whether the narrated event occurs before, during, or after the speech time [past, present, or future event; (Reichenbach, 1947)]. In many languages, notably tensed languages (e.g., English or French), time reference is made through verb inflection (i.e., inflectional morphemes attached to the verb) and adverbs of time (Grisot, 2018). Morphosyntactic difficulties, such as those observed in aphasia, are therefore likely to impair the ability to produce time reference through verbal inflection and to set the temporal framework of discourse, with a significant impact on daily communication.

Several studies have reported difficulties in tense marking in people with aphasia. However, not all tenses are similarly affected, and some dissociations between tense categories (i.e., past vs. present vs. future) have been found. Thus, this latter observation led Bastiaanse et al. (2011) to develop the PAst DIscourse LInking Hypothesis (PADILIH). According to the PADILIH, past tense is selectively impaired due to its discourse-linked nature. Indeed, since the event time is prior and therefore dissociated from the speech time, it is necessary to establish a discourse link between these two points in time. Such a link is costly in terms of processing resources, leading to difficulties in producing past tense. On the other hand, present tense does not require this discourse-linking as the event time coincides with the speech time. It is therefore preserved. Similarly, in the case of the future tense, the event has not yet taken place and cannot, therefore, be referred to. Consequently, the future tense is also not linked to discourse and represents a subclass of the present.

This specific past tense deficit has been supported by several experimental studies, most of which were carried out on people with non-fluent agrammatic aphasia. These studies have shown poorer performance in past tense compared to present and future tenses (Bastiaanse, 2008, 2013; Yarbay Duman and Bastiaanse, 2009; Dragoy and Bastiaanse, 2013; Martínez-Ferreiro and Bastiaanse, 2013; Rofes et al., 2014). Some studies have suggested that this performance profile may also be found in people with fluent aphasia, albeit with different error patterns (Jonkers and de Bruin, 2009; Dragoy and Bastiaanse, 2013; Bos and Bastiaanse, 2014). However, some studies have also reported contradictory results to the PADILIH, with no dissociation found between past, present and future tenses (Faroqi-Shah and Thompson, 2004, 2007; Burchert et al., 2005; Varlokosta et al., 2006; Clahsen and Ali, 2009; Faroqi-Shah and Dickey, 2009; Fyndanis et al., 2012).

Several explanations could account for these discrepancies. Firstly, the type of task used in the studies could influence the results. Indeed, the PADILIH was mainly supported by one task, the Test for Assessing Reference of Time (TART) (Bastiaanse et al., 2008). This task involves transposing the structure of a sentence given for the first action depicted in a picture to a second, slightly different action depicted in another picture [e.g.*, For this photo, I could say, “Previously, the man peeled an apple”; for this picture* (showing a man with an empty plate), *you might say “Previously, the man…”* (expected answer: ate an apple)] (Bastiaanse et al., 2011). This task could thus increase the demand on working memory in the past condition, as the action is not represented in the picture. By contrast, in the present condition, the photographs show the action in progress (Faroqi-Shah and Friedman, 2015). Furthermore, the TART would predominantly involve the retrieval component of the time reference process (i.e., retrieving the phonological form of the verb – *ate* in the example) (Fyndanis et al., 2018a). The encoding component (i.e., the diacritic feature of past) is less solicited as the transposition implies that the participant remains in the same temporal frame. Other tasks, such as those involving transformation from one time reference feature to another based on a temporal adverbial (e.g., *Yesterday, the man ate an apple. Tomorrow, the man…* (expected response: will eat) *an apple*) involve both components. They are, therefore, likely to be more difficult, leading to deficits in several tenses (Nanousi et al., 2006; Fyndanis et al., 2012, 2018a).

Secondly, factors inherent to the participants could also influence their performance and partly explain inter-study differences. In particular, an age-related decline in grammatical processing has been reported, with reduced production of complex sentences and greater difficulties in producing subject-verb agreements in older adults [see (Marini and Andreetta, 2016) for a review]. As tense is generally more complex than agreement (Nanousi et al., 2006; Varlokosta et al., 2006; Kok et al., 2007), age also likely impacts time reference. These difficulties could be linked to working memory capacity, which tends to decline with age (Klencklen et al., 2017). Indeed, tense production is costly in working memory. Producing the correct verb form involves activating conceptual information and morphosyntactic features and then retrieving the corresponding verbal form (Fyndanis et al., 2018b). This cost can also be exacerbated in specific tasks, such as transformation tasks, which require the verbal form of the source sentence to be inhibited. Several studies have thus demonstrated a link between working memory and tense in people with aphasia and healthy people (Kok et al., 2007; Fyndanis et al., 2018b). The effect of age on time reference, however, has been little studied. To our knowledge, only two studies (Fyndanis et al., 2018b; Coulombe et al., 2019) have analyzed this link, with contradictory results. The complexity of the tasks used might be at the root of these discrepancies.

Another variable of interest is gender. A study with a large sample of people with aphasia showed a gender effect to the disadvantage of men on several language measures (e.g., sentence completion or listening comprehension) (Sharma et al., 2019). However, the effect of gender on tense production has not yet been studied. If such an effect exists, the discrepancies between studies could be partly explained by the characteristics of their participants.

Finally, the classification of the future as a subcategory of the present is not unanimous. Some researchers have postulated that the future, like the past, may be discourse-linked, as it, too, requires a link to be established between the event time (in the future) and the speech time (Avrutin, 2000). Consequently, the questions of whether one tense is more impaired than another in fluent and non-fluent aphasia and whether these potential dissociations can be explained by some variables (e.g., tasks, socio-demographics) remain unanswered.

A previous meta-analysis was conducted by Faroqi-Shah and Friedman (2015) to study the questions of dissociation within tenses in non-fluent aphasia and task effect. Their results revealed significant differences between the past and present tenses. The future tense did not differ from the past and present tenses. When analyses were performed separately for each task, only one task, sentence production priming (i.e., the TART), showed poorer performance for the past and future tenses compared with neutral nonfinite sentences. The difference between the past and present tenses was marginally significant. However, unlike the PADILIH prediction, the past and future tenses did not differ significantly. No significant difference between tenses was observed in the other tasks, namely sentence production using picture description with temporal adverbs as prompts, grammaticality judgment, and sentence completion based on a temporal adverb with forced choice. The authors concluded that there was a task effect on time reference. However, this meta-analysis only included participants with non-fluent aphasia. Therefore, it is not possible to draw any conclusions about the performance pattern of people with fluent aphasia. Moreover, the influence of socio-demographic variables was not considered in this meta-analysis.

The present study thus aims to characterize tense impairment precisely, in people with fluent and non-fluent aphasia through a systematic review and individual participant data (IPD) meta-analysis. More specifically, it aims to answer the two following questions:

- To what extent is one tense more impaired than other tenses (past, present, future) in speakers with fluent and non-fluent aphasia?
- Which factors (related to the task and the speaker) moderate tense effects?

## 2 Method

This research is based on the Preferred Reporting Items for Systematic Reviews and Meta-Analysis (PRISMA) guidelines (Page et al., 2021). A systematic review was first performed, followed by an IPD meta-analysis. The choice of electronic databases, search strategy, study selection, data extraction, and statistical analysis was guided by our research questions.

Our protocol has not been registered or published on any platform.

### 2.1 Data sources and search strategy

Three electronic databases, Ovid-MEDLINE (1946 to March 2022), Scopus (1960 to March 2022), and ProQuest-APA PsycInfo (1967 to March 2022), were explored in March 2022. Keywords related to aphasia and verbal inflection were used. The search strategy applied in the Ovid interface (MEDLINE) is available in Table 1. The search strategy was adapted using mesh terms and truncators for the specificities of each database.

**Table 1:** Search strategy.

Search strategy
Aphasia/ agrammati*.ti,ab,kf. inflect*.ti,ab,kf. “time reference.”ti,ab,kf. tense.ti,ab,kf. morphology*.ti,ab,kf. 1 or 2 3 or 4 or 5 or 6 7 and 8 limit 9 to yr. = “1994-Current”

### 2.2 Study selection

On 8th March 2022, the first literature search was performed in the three databases and updated on 28th March 2023. All the references were exported into an Excel data sheet, and one investigator (ES) removed the duplicate records. The initial screening was then performed by two independent investigators (NC and ES). Title and abstract were screened for each record yielded by the literature search, and irrelevant studies were excluded according to the eligibility criteria available in Table 2. Then, full texts of the remaining records were read by the same investigators, and the same criteria were used to include or exclude the articles.

**Table 2:** Eligibility criteria.

Eligibility criteria for the qualitative review		
	Inclusion criteria	Exclusion criteria
Population	Adult with a vascular aphasia Fluent or non-fluent aphasia Monolingual	Aphasia following a traumatic brain injury, a tumor, a neurodegenerative disease Bilingual
Intervention	At least one task assessing verbal inflection in production in a sentence context (constraint task) At least a comparison of two tenses (past, present, future)	Tasks assessing verbal inflection in discourse, semi-spontaneous speech, picture description, comprehension, reading, repetition Only one tense is assessed
Publication type	Peer-reviewed publication Written in English or French Original contributions to the literature Group or case study Publication year after 1994	(Systematic) reviews or meta-analysis studies Book chapters Conference reports Thesis Written in other languages

Eligibility criteria for the individual participant data meta-analysis		
	Inclusion criteria	Exclusion criteria
Results	Individual data are available Individual data are sent by the authors	No access to individual data
Number of stimuli per condition	At least 10 items for each tense condition	Less than 10 items per tense condition
Quality assessment	Study quality rated as good or fair	Study quality rated as poor

Disagreements on the inclusion of a record were resolved by discussion between the two investigators for both steps of the selection process.

### 2.3 Data extraction

Relevant data were extracted by two independent reviewers (NC and ES) in a data table. Extracted data were classified into five main categories: (1) study characteristics (e.g., authors, year of publication, title, study goal), (2) population characteristics (e.g., sample size, aphasia type, fluency type, language), (3) methodological characteristics (e.g., task type, tenses assessed, number of items in each condition, score calculated, type of errors analysis, availability of individual data, statistical analysis), (4) main results, (5) limits of the study.

### 2.4 Quality assessment

The quality of included studies was assessed using the Appraisal tool for Cross-Sectional Studies (AXIS) (Downes et al., 2016). This tool was chosen because of its suitability to our study and its relevance, which had already been recognized by several researchers (Ma et al., 2020; Moskalewicz and Oremus, 2020). It contains 20 questions concerning five parts of a study: introduction (1 question on study objectives), methods (10 questions; e.g., justification and description of the participant sample, measures and statistical method used), results (5 questions; e.g., internal consistency of results, descriptive statistics), discussion (2 questions; discussion of all results and limitations), and other (2 questions; funding/conflicts of interest and ethics). Questions 7, 13, and 14, dealing with non-responders (description, categorization, rate), were removed as they did not apply to our study designs. Three independent investigators (NC, LZ, and ES) used the 17 remaining questions to assess the quality of each included study, answering each of them with “Yes,” “No,” or “Do not know.” Points were given to each question according to the answer: 2 points for a “Yes,” 1 point for a “Do not know” and 0 points for a “No.” Disagreements on the answers were resolved by discussion between the investigators.

If the AXIS tool (Downes et al., 2016) guides the investigator in how to assess study quality, it does not provide a numerical scale to assess it. Therefore, a quality score was calculated for each study by summing all the question points, with a maximum of 34 points. According to the percentage of points obtained, the study quality was then rated as good (> 75%), fair (50–75%), or poor (<50%). These criteria were chosen following the literature (e.g., Grimm et al., 2021; Setién-Suero et al., 2022). Only studies rated as fair or good were included in the IPD meta-analysis.

The AXIS tool (Downes et al., 2016) was also used to assess the quality of the case studies meeting the inclusion criteria. The same procedure as the one explained for cross-sectional studies was used. However, questions 3 and 6 (i.e., justification and representativeness of sample size) were removed as they did not apply to case reports (maximum of points = 30). The same tool as for cross-sectional studies was chosen for case reports because it enabled us to have the same criteria in quality assessment. Furthermore, if an assessment scale exists for case reports (JBI for Case report; The Joanna Briggs Institute, 2019), the questions provided did not apply to the two case reports included in our study.

### 2.5 Statistical analysis

Traditional meta-analyses use a “two-stage” approach, calculating the effect size in each study and then combining them. Our meta-analysis is different, however, in that we had direct access to the individual participant data from each study. Therefore, in line with the recommendations from Simmonds and Higgins (2016), we used a “one-stage” IPD meta-analysis or mega-analysis approach (Eisenhauer, 2021). The advantage of this one-stage approach is that it does not rely on the assumption that effect estimates are normally distributed or that standard errors are known, assumptions that are less valid in the case of small studies such as in our meta-analysis (Simmonds and Higgins, 2016). In addition, the IPD meta-analysis allows sources of variance to be better taken into account (Koile and Cristia, 2021).

For both study’s objectives, we used mixed-effects logistic regression models corresponding to random-effect models with moderators in a meta-analysis, with two levels of variability (studies and participants). They were conducted on the binary (correct/incorrect) responses of each participant, using R (R Development Core Team, 2008) and the *lme4* package (Bates et al., 2015). We allowed the studies and participants to vary with respect to their intercepts and slopes (where relevant, see below) by adding them as random effect terms in our models. Likelihood ratio tests were systematically used to compare the models with the main effect or interaction to a model without it to assess the significance of the main effects and interactions (Pinheiro and Bates, 2000). Note that in logistic models, estimates (β) are measured on a logit scale. Visual inspection of residuals and regression diagnostics from the DHARMa package (Hartig, 2022) showed no violation of model assumptions. The R scripts used for the analysis and the Excel sheet with all individual data are provided in Supplementary materials S1, S2.

For the first objective, our model included fixed effects for tense, aphasia fluency, and the interaction between tense and aphasia fluency. For the second objective, additional models were run to test the tense effect in interaction with the task, and the tense effect in interaction with age and gender. Post-hoc tests were performed with Tukey correction for multiple comparisons using “lsmeans” package (Lenth, 2016). The significance level was set at *p* < 0.05.

Heterogeneity was determined through comparisons of models with and without the different sources of variance (intercepts for subjects and studies, by subject, and by studies random slopes for the effect of tense) with likelihood ratio tests. A significant difference in favor of the models with the sources would attest to the presence of heterogeneity and thus justify using random effect rather than fixed effect models.

Publication bias was assessed graphically with funnel plots of average study effect sizes against their standard error. Statistical tests for funnel plot asymmetry were not performed as we had two sources of variance (participants and studies) and fewer than 10 studies for fluent participants (Sterne et al., 2011).

## 3 Results

**Figure 1:**
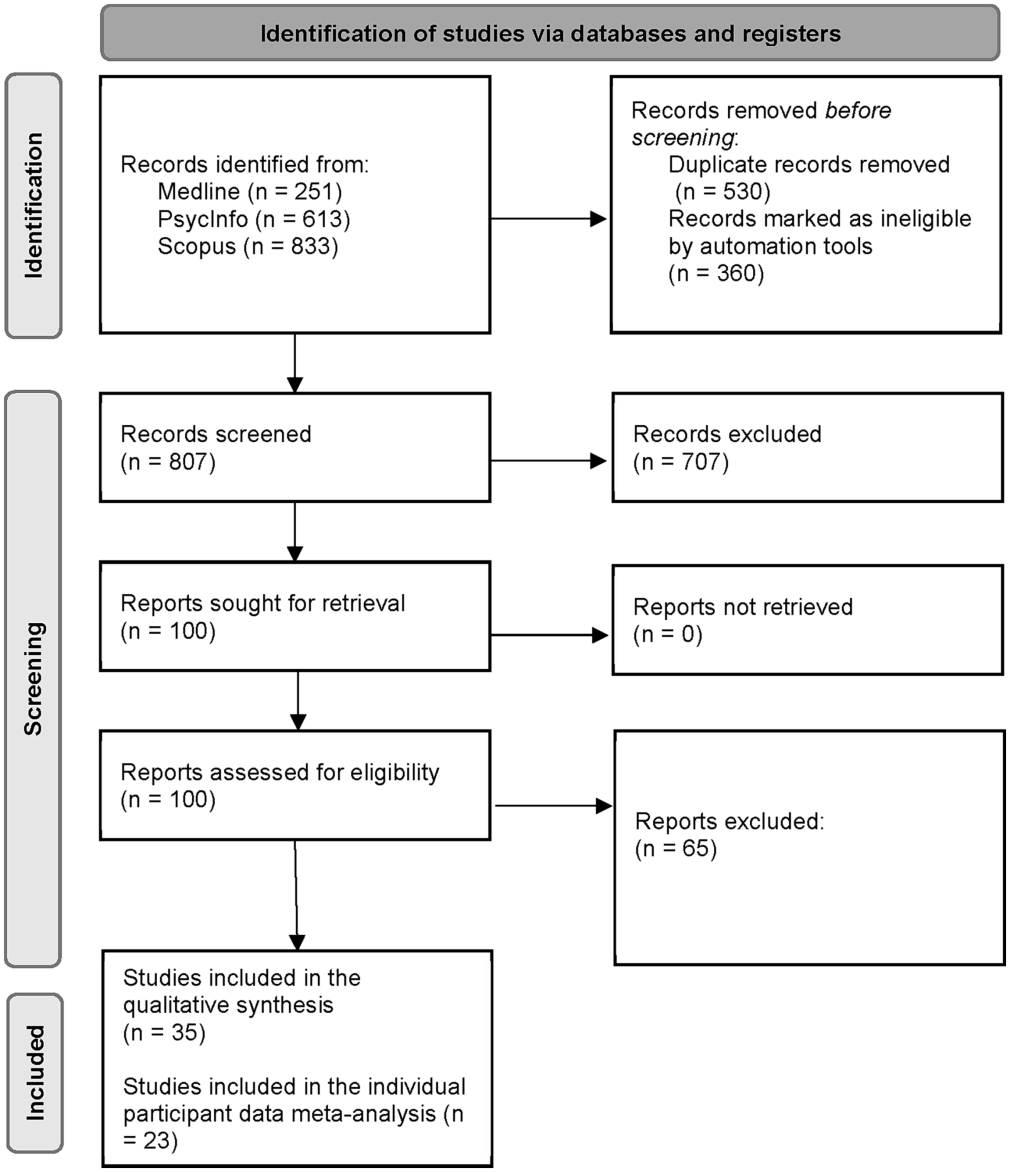
Flow diagram of the identification and selection of records, adapted from the PRISMA flow diagram (Page et al., 2021).

The initial literature search in the three electronic databases yielded 1,646 records, and the updated literature search resulted in 51 new records. Five hundred and thirty duplicates and 360 records marked as ineligible by automation tools were removed, resulting in 807 records. The screening for titles and abstracts resulted in 100 records. After full-text reading, 35 articles met the inclusion criteria for the qualitative synthesis, and 23 studies (232 participants, 45 with fluent aphasia and 187 with non-fluent aphasia) met the inclusion criteria for the IPD meta-analysis (see Figure 1).

In the following sections, we first report general characteristics of the studies included in the qualitative synthesis and then describe the results from the statistical analysis performed for the IPD meta-analysis.

### 3.1 Qualitative synthesis

The main characteristics of the 35 studies included in the qualitative synthesis are summarized in Table 3.

**Table 3:** Main characteristics of the studies included in the qualitative synthesis.

Study	Population				Experimental tasks	Results				
	Sample			Language		Percentage of correct answers			Statistical differences	Error analysis
	Fluent	Non-fluent	Control			Past	Present	Future		
Aithal et al. (2009)	–	11	–	Malayalam	Picture	76	94	33	NR	No
Alqhazo and Abu (2021)	–	20	20	Jordanian Arabic	ADV-MC	NR	NR	NR	Past < present, future*	Yes
Auclair-Ouellet et al. (2019) – exp. 1	1	–	5	Swiss-German	ADV	40	20	17	NR NR	Yes
– exp. 2	1	–	5	Swiss-German	ADV-MC	77	90	75		Yes
Bastiaanse (2008)	–	10	10	Dutch	ADV	39	56	-	Past < present*	Yes
Bastiaanse (2011)	– – –	11 12 8	30	Chinese English Turkish	TART	32 50 44	31 78 72	36 70 80	Non-significant past < Present = future* past < present = future*	Yes
Bos and Bastiaanse (2014)	16	24	20	Dutch	TART	45	68	–	Non-significant	Yes
Burchert et al. (2005)	–	9	9	German	TS-MC	69	67	–	Non-significant	Yes
Dragoy and Bastiaanse (2013)	7	7	7	Russian	TART	56	77	51	Effect of time reference	Yes
El Ouardi (2021)	-	5	5	Moroccan Arabic	TS-MC	42	74	68	Past < present = future*	Yes
Faroqi-Shah and Friedman (2015)	–	16	–	English	ADV	19	22	30	Non-significant	Yes
Faroqi-Shah and Thompson (2004)	–	8	–	English	ADV	NR	NR	NR	Non-significant	Yes
Faroqi-Shah and Thompson (2007) – exp. 2	–	10	–	English	ADV-MC	55	53	–	Non-significant NR	Yes
– exp. 3	–	9	–	English	ADV-MC	45	54	53		Yes
Fyndanis et al. (2018a)	5 –	5 7	21	Greek Italian	TS	66 69	– –	70 76	Non-significant Non-significant	Yes
Fyndanis and Themistocleous (2019)	4	4	103	Greek	TS	62	–	70	NR	No
Fyndanis et al. (2012)	-	2	2	Greek	TS	76	38	45	NR	No
Jonkers and De Bruin (2009)	5	7	20	Dutch	ADV	52	76	–	Past < present*	Yes
Kok et al. (2007)	–	9	9	Dutch	ADV	55	45	–	NR	Yes
Koukoulioti et al. (2020)	8	2	10	Greek	ADV	72	70	-	Non-significant	Yes
Koukoulioti and Bastiaanse (2019)	8	2	10	Greek	TART	64	85	70	Past and future < present*	No
Kurada et al. (2022)	–	17	17	Turkish	TART	24	93	89	Past < non-past*	Yes
Lee et al. (2013)	–	5	9	Korean	TART	40	55	51	Non-significant	Yes
Lee et al. (2008) – exp. 2	–	4	4	English	ADV	35	30	–	Non-significant	Yes
Martínez-Ferreiro and Bastiaanse (2013)	–	3 7	NR	Spanish Catalan	TART	53	65	63	Past < present = future*	Yes
Marusch et al. (2017)	–	5	5	English	TART	NR	NR	–	Past < present*	Yes
Marusch et al. (2012)	–	9	7	German	TART	NR	NR	–	Past < present*	Yes
Miozzo et al. (2010)	–	1	5	English	TS	74	25	–	NR	No
Nanousi et al. (2006) – task 3	–	6	NR	Greek	TS	41	49	41	NR NR	Yes
– task 5	–	6	Greek	TS	58	72	53	NR		Yes
Nerantzini et al. (2020)	–	7	7	Greek	TART	32	86	56	Past < present*	Yes
Patterson and Holland (2014)	–	4	–	English	ADV	53	64	–	NR	Yes
Rofes et al. (2014)	–	9	9	Catalan	TART	22	–	53	Past < future*	Yes
Siriboonpipattana et al. (2020)	–	12	18	Thai	TART	81	77	62	NR	Yes
Thompson et al. (2013) – exp. 2	9	20	–	English	ADV	64	64	–	NR	No
Tsiwah et al. (2021)	–	7	10	Akan	TART	56	80	88	Past < present, future*	Yes
Wenzlaff and Clahsen (2004)	–	7	7	German	ADV-MC	67	69	–	NR	No
Yarbay Duman and Bastiaanse (2009)	–	7	7	Turkish	TS	37	–	51	Tensed: past < future Participle: past = future	Yes

*Experimental task*: ADV: sentence completion task according to a temporal adverb; ADV-MC: sentence completion task according to a temporal adverb with multiple choice answers; Picture: picture description; TART: Test for Assessing Reference of Time (Bastiaanse et al., 2008); TS: transformational sentence completion task; TS-MC: transformational sentence completion task with multiple choice answers; *Other*: NR: not reported; *significant difference.

Concerning the population, most of these studies involved participants with non-fluent aphasia (34 of the 35 studies, for a total of 328 participants), nine studies involved participants with fluent aphasia (64 participants), and 29 studies involved a control group (391 participants; two studies did not report information concerning a control group). Time reference was assessed in these participants in 17 languages. English was the most frequently assessed language in eight studies, followed by Greek in seven studies, Dutch in four studies, German and Turkish in three studies, Catalan in two studies, and Akan, Jordanian Arabic, Moroccan Arabic, Chinese, Italian, Korean, Malayalam, Russian, Spanish, Swiss-German, and Thai in one study.

Regarding the experimental tasks, six types of task were used to assess time reference (see Supplementary materials S3 for examples). The Test for Assessing Reference of Time (TART) (Bastiaanse et al., 2008) was used in 13 studies, a sentence completion task according to a temporal adverb was used in 10 studies, a transformational sentence completion task was used in six studies, a sentence completion task according to a temporal adverb with multiple choice answers was used in four studies, a transformational sentence completion task with multiple choice answers was used in two studies, and a picture description was used in one study. All the included studies assessed time reference for at least two tenses, and the past was assessed in all of these studies. The three tenses, past, present, and future, were explored in 18 studies. Eighteen studies assessed time reference in two tenses, 14 in past and present, and 4 in past and future.

Of the 35 studies, 24 statistically analyzed differences between tenses. Twelve studies (Bastiaanse, 2008; Jonkers and de Bruin, 2009; Bastiaanse et al., 2011; Marusch et al., 2012, 2017; Martínez-Ferreiro and Bastiaanse, 2013; Rofes et al., 2014; Nerantzini et al., 2020; Alqhazo and Abu, 2021; El Ouardi, 2021; Tsiwah et al., 2021; Kurada et al., 2022) found that people with aphasia have more difficulties producing one tense compared to the other assessed tenses: the past was more altered than the present and/or the future in 11 studies, and the future was more altered than the present in one study (Koukoulioti and Bastiaanse, 2019). One study (Dragoy and Bastiaanse, 2013) did not detail the effect of time, and no study found that present was the most impaired tense. However, 8 studies (Faroqi-Shah and Thompson, 2004; Burchert et al., 2005; Lee et al., 2008, 2013; Bos and Bastiaanse, 2014; Faroqi-Shah and Friedman, 2015; Fyndanis et al., 2018a; Koukoulioti et al., 2020) found no difference between tenses (3 explored the three tenses, 4 explored past and present, and 1 study explored past and future). Two studies (Faroqi-Shah and Thompson, 2007; Bastiaanse et al., 2011) had mixed results depending on the language assessed or the task used. Eleven studies (Wenzlaff and Clahsen, 2004; Nanousi et al., 2006; Kok et al., 2007; Aithal et al., 2009; Miozzo et al., 2010; Fyndanis et al., 2012; Thompson et al., 2013; Patterson and Holland, 2014; Auclair-Ouellet et al., 2019; Fyndanis and Themistocleous, 2019; Siriboonpipattana et al., 2020) did not statistically analyze tenses differences. Therefore, quantitative analysis in the IPD meta-analysis will provide more insight into these results.

Twenty-eight studies analyzed the types of errors made by people with aphasia. The most reported error categories were substitutions of one tense for another (e.g., *changes* for *changed*), use of unmarked forms (e.g., infinitives as *change*), omissions (of an auxiliary, for example), and other errors (e.g., no answer, agreement errors, neologisms). However, it should be noted that the definition of these categories, and the type of errors corresponding, often vary from one study to another. This variation is partly explained by the languages studied, with some error categories being more relevant in certain languages. For example, omissions are more frequently encountered in Chinese since tense is marked by aspectual adverbs rather than inflection (Bastiaanse et al., 2011).

#### 3.1.1 Quality assessment

The results of the quality assessment for the 35 studies included in the qualitative synthesis are shown in Table 4. Three studies were rated good and 31 fair. The study of Lee et al. (2008) was the only one assessed as having poor quality and was therefore excluded from the IPD meta-analysis.

**Table 4:** Quality assessment of studies included in the systematic review using the appraisal tool for cross-sectional studies (AXIS) (Downes et al., 2016).

Study	Intro	Methods									Results			Discussion		Other		Score
	Q1	Q2	Q3	Q4	Q5	Q6	Q8	Q9	Q10	Q11	Q12	Q15	Q16	Q17	Q18	Q19	Q20	
Aithal et al. (2009)	Yes	Yes	No	Yes	DK	Yes	No	Yes	Yes	Yes	No	DK	Yes	Yes	Yes	DK	DK	FAIR
Alqhazo and Abu (2021)	Yes	Yes	No	Yes	Yes	No	Yes	Yes	Yes	Yes	No	Yes	No	DK	Yes	Yes	Yes	FAIR
Auclair-Ouellet et al. (2019)	Yes	Yes	NA	Yes	DK	NA	Yes	No	No	No	Yes	Yes	DK	Yes	No	Yes	DK	FAIR
Bastiaanse (2008)	Yes	Yes	No	Yes	DK	No	Yes	Yes	No	No	Yes	Yes	Yes	Yes	No	DK	DK	FAIR
Bastiaanse et al. (2011)	Yes	Yes	No	Yes	DK	No	Yes	Yes	No	No	Yes	No	Yes	Yes	No	DK	DK	FAIR
Bos and Bastiaanse (2014)	Yes	Yes	No	Yes	DK	No	Yes	Yes	Yes	Yes	Yes	Yes	Yes	Yes	No	Yes	Yes	GOOD
Burchert et al. (2005)	Yes	Yes	No	Yes	DK	No	Yes	No	No	No	Yes	Yes	DK	No	No	Yes	DK	FAIR
Dragoy and Bastiaanse (2013)	Yes	Yes	No	Yes	DK	Yes	Yes	Yes	No	No	Yes	Yes	DK	Yes	Yes	Yes	DK	FAIR
El Ouardi (2021)	Yes	Yes	No	Yes	DK	Yes	Yes	Yes	No	No	Yes	DK	No	Yes	Yes	Yes	Yes	FAIR
Faroqi-Shah and Friedman (2015)	Yes	Yes	No	Yes	DK	No	Yes	No	No	No	Yes	No	DK	Yes	No	Yes	DK	FAIR
Faroqi-Shah and Thompson (2004)	Yes	Yes	No	Yes	DK	Yes	Yes	Yes	No	No	Yes	No	Yes	Yes	Yes	Yes	DK	FAIR
Faroqi-Shah and Thompson (2007)	Yes	Yes	No	Yes	Yes	No	Yes	No	No	No	Yes	Yes	DK	Yes	No	Yes	DK	FAIR
Fyndanis et al. (2018a)	Yes	Yes	No	Yes	DK	No	Yes	No	No	No	Yes	Yes	Yes	Yes	No	Yes	Yes	FAIR
Fyndanis and Themistocleous (2019)	Yes	Yes	No	Yes	DK	Yes	Yes	No	No	No	Yes	Yes	Yes	Yes	No	Yes	Yes	FAIR
Fyndanis et al. (2012)	Yes	Yes	No	Yes	DK	Yes	Yes	No	No	No	Yes	Yes	DK	Yes	No	DK	DK	FAIR
Jonkers and De Bruin (2009)	Yes	Yes	No	Yes	DK	No	Yes	No	No	No	Yes	Yes	DK	Yes	No	DK	DK	FAIR
Kok et al. (2007)	Yes	Yes	No	Yes	DK	Yes	Yes	No	No	No	Yes	Yes	No	Yes	No	DK	DK	FAIR
Koukoulioti et al. (2020)	Yes	Yes	No	Yes	DK	Yes	Yes	No	No	No	Yes	Yes	Yes	Yes	No	Yes	DK	FAIR
Koukoulioti and Bastiaanse (2019)	Yes	Yes	No	Yes	DK	No	DK	Yes	No	No	No	DK	DK	Yes	No	Yes	Yes	FAIR
Kurada et al. (2022)	Yes	Yes	No	Yes	Yes	Yes	Yes	Yes	Yes	Yes	Yes	DK	Yes	Yes	No	Yes	Yes	GOOD
Lee et al. (2013)	Yes	Yes	No	Yes	Yes	Yes	Yes	Yes	No	No	Yes	Yes	DK	Yes	No	DK	DK	FAIR
Lee et al. (2008)	Yes	Yes	No	Yes	DK	No	Yes	No	No	No	No	Yes	DK	No	No	Yes	DK	POOR
Martínez-Ferreiro and Bastiaanse (2013)	Yes	Yes	No	Yes	Yes	Yes	Yes	Yes	No	No	Yes	Yes	Yes	Yes	No	Yes	DK	FAIR
Marusch et al. (2017)	Yes	Yes	No	Yes	DK	Yes	Yes	Yes	No	No	No	DK	No	Yes	No	Yes	DK	FAIR
Marusch et al. (2012)	Yes	Yes	No	Yes	DK	Yes	Yes	Yes	No	No	No	DK	No	Yes	No	Yes	Yes	FAIR
Miozzo et al. (2010)	Yes	Yes	NA	Yes	DK	NA	DK	No	No	No	Yes	Yes	DK	Yes	No	Yes	DK	FAIR
Nanousi et al. (2006)	Yes	Yes	No	Yes	DK	Yes	No	Yes	No	No	No	DK	DK	Yes	No	Yes	DK	FAIR
Nerantzini et al. (2020)	Yes	Yes	No	Yes	DK	Yes	Yes	Yes	No	No	Yes	Yes	DK	Yes	No	Yes	DK	FAIR
Patterson and Holland (2014)	Yes	Yes	No	Yes	DK	No	Yes	Yes	No	No	Yes	DK	DK	Yes	No	DK	DK	FAIR
Rofes et al. (2014)	Yes	Yes	No	Yes	DK	Yes	Yes	No	No	No	Yes	Yes	Yes	Yes	No	Yes	DK	FAIR
Siriboonpipattana et al. (2020)	Yes	Yes	No	Yes	Yes	No	Yes	Yes	No	No	Yes	Yes	Yes	Yes	No	Yes	Yes	FAIR
Thompson et al. (2013)	Yes	Yes	No	Yes	Yes	Yes	Yes	Yes	No	No	Yes	DK	Yes	Yes	No	DK	Yes	FAIR
Tsiwah et al. (2021)	Yes	Yes	No	Yes	Yes	Yes	Yes	Yes	Yes	Yes	Yes	Yes	Yes	Yes	No	Yes	Yes	GOOD
Wenzlaff and Clahsen (2004)	Yes	Yes	No	Yes	DK	Yes	Yes	No	No	No	Yes	Yes	DK	Yes	No	Yes	DK	FAIR
Yarbay Duman and Bastiaanse (2009)	Yes	Yes	No	Yes	Yes	No	Yes	No	No	No	Yes	No	DK	Yes	No	Yes	DK	FAIR

Questions 7, 13, and 14 were not applicable for our studies and were removed. DK = Do not know; NA = Not applicable.

All studies stated a clear aim for their research (Q1) and used an appropriate design for the stated aim (Q2). For the method section, the quality assessment shows some weaknesses. For the population, none of the included studies justified their sample size (Q3), and 25% of the included studies gave information about where the participants were recruited (Q5). Concerning the design and the statistical analysis, 57% of the studies correctly measured the risk factor or the outcome variables of their material (Q9), and 14% clearly stated what they used to determine the statistical significance or sufficiently described their analysis method (Q10 and Q11). For the results section, 63% of the studies reported internally consistent results (Q15), and 40% of the studies reported results for all the analyses described in the method section (45% of the studies were classified as “Do not know” as the statistical method was often not clearly described) (Q16). Finally, 91% of the discussion and conclusion of the included studies were justified by their results (Q17), and 14% of the studies discussed some of the limitations of their study (Q18). Information concerning participants’ ethical approval or consent was given in 31% of the studies (Q20).

### 3.2 Individual participant data meta-analysis

#### 3.2.1 Objective 1. Effect of tense in speakers with fluent and non-fluent aphasia

**Figure 2:**
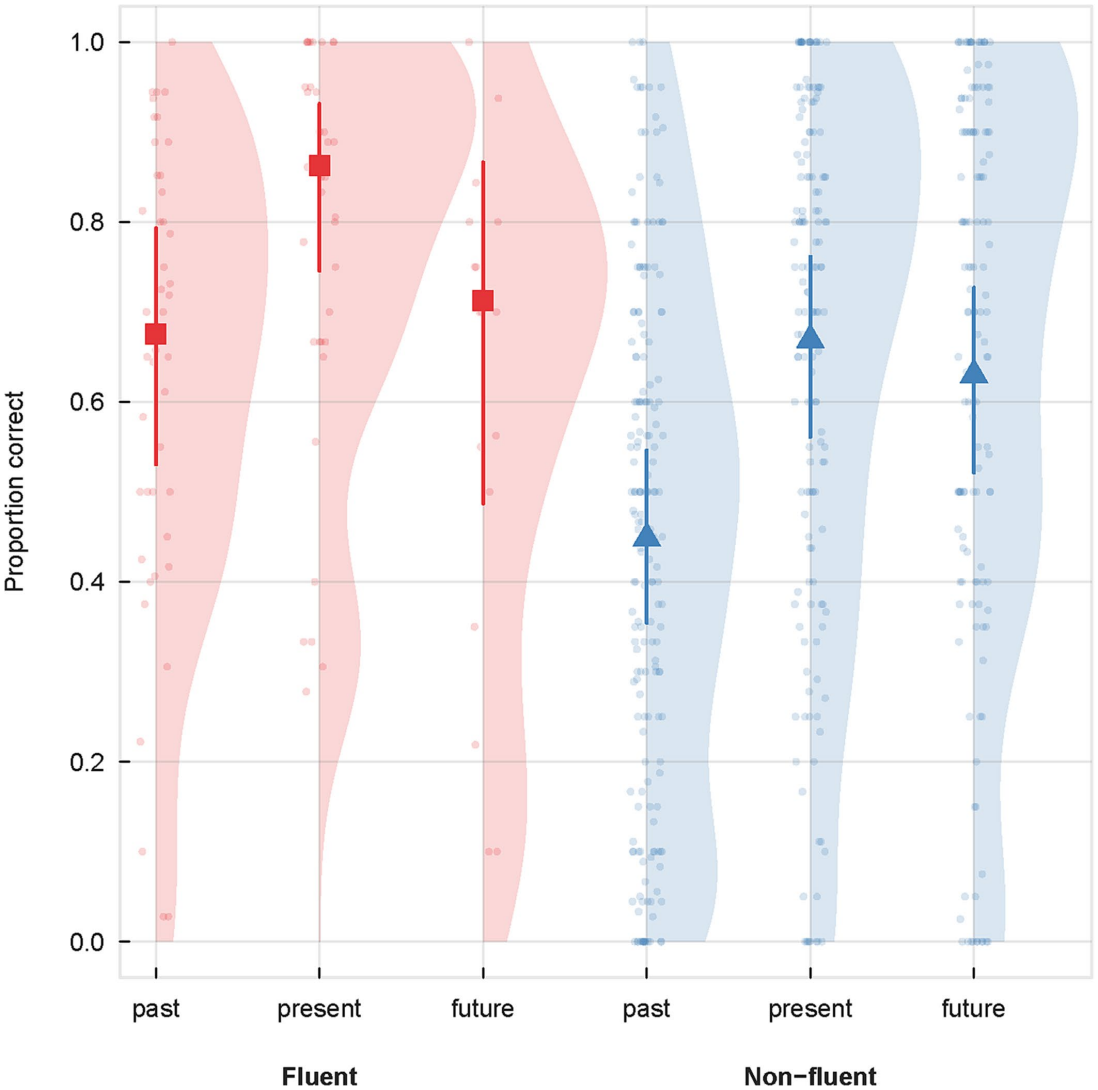
Percentage of correct responses for participants with fluent and non-fluent aphasia for each tense (e.g., past, present, and future) with data distribution. The model predictions and raw data for the proportion of correct answers as function of tense (past, present and future) and participant fluency (fluent and non-fluent aphasia). The squares and triangles with arrows depict the (fixed effect) model predictions and 95% confidence intervals of the mixed logistic regression across the three tenses for fluent and non-fluent participants, respectively. The faint small dots show the raw data, i.e., the average proportion answered correctly for each participant. The shaded distribution represent the Kernel density of the participant means for the six tense/fluency combinations.

**Figure 3:**
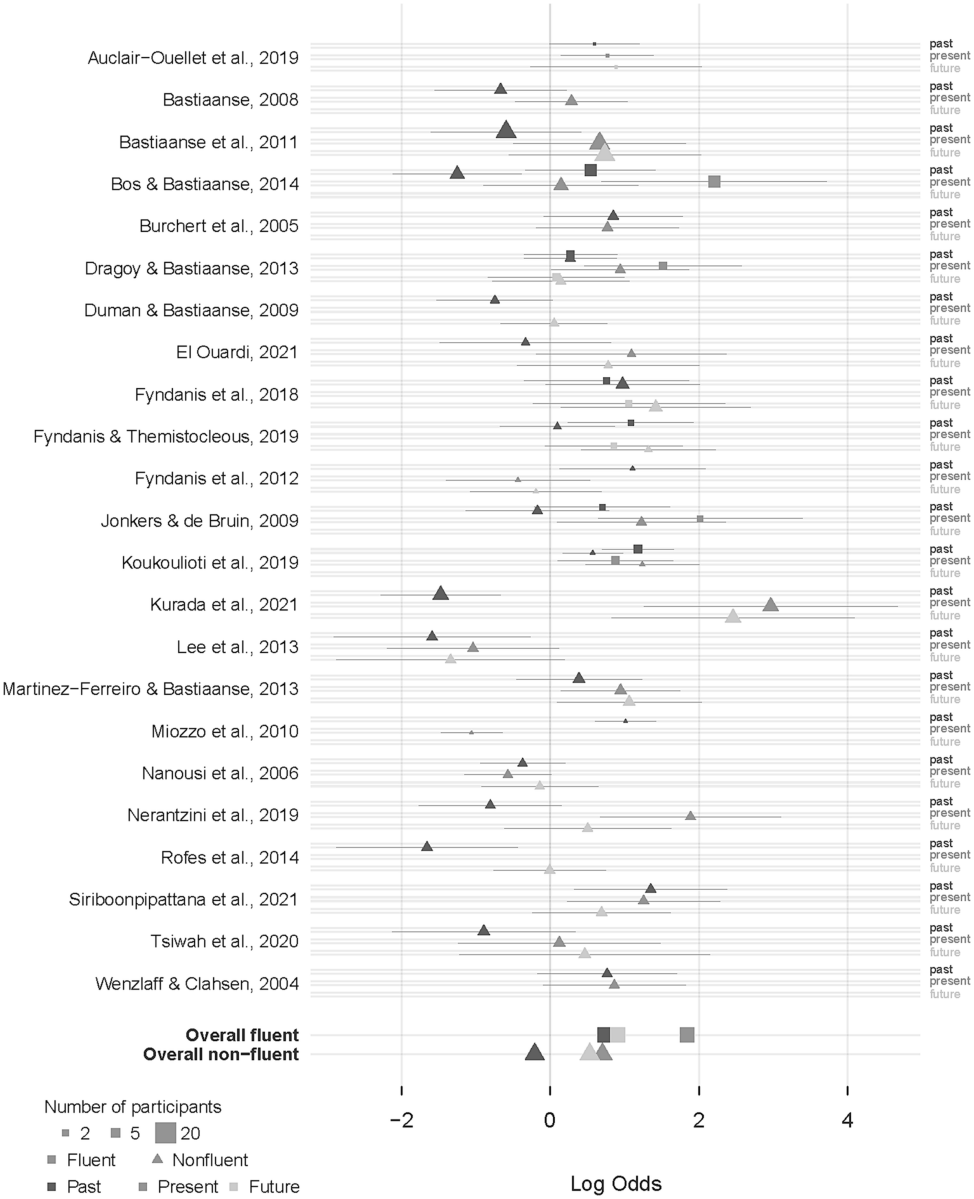
Forest plots showing effect size estimates (log odds) for participants with non-fluent and fluent aphasia in each tense (past, present and future). Log-odds model predictions and 95% predictive intervals of the proportion correct for past, present and future tenses and participant fluency (squares for fluent aphasia and triangles for non-fluent aphasia) for all the articles used in this study. The size of the squares/triangles is proportional to the sample size in the respective study and tense/fluency combination. The overall (fixed) effects across all studies for the three tenses and two fluency levels are shown at the bottom.

Figure 2 shows the mixed effect model predictions of percentage of correct responses for participants with fluent and non-fluent aphasia for each tense (e.g., past, present, and future). Figure 3 summarizes the study-level predictions of the IPD meta-analysis using a mixed-effects model.

Likelihood ratio tests revealed that the model with the most complex random effect structure (random slope and intercept for study and random intercept for subject) fitted the data significantly better than the models without these variability sources (see Supplementary materials S4). This result attests to heterogeneity between studies and subjects and justifies the use of mixed-effects models for the meta-analysis.

Likelihood ratio tests (see also Supplementary materials S5a for the results of the model) revealed a significant effect of the tense [*χ*^2^(2) = 7.07, *p* = 0.029] and the aphasia fluency [*χ*^2^(1) = 9.07, *p* = 0.003] but no significant effect of the interaction between the tense and aphasia fluency [*χ*^2^(2) = 2.28, *p* = 0.321]. The significant effect of tense showed that the probability of answering correctly is lower in the past tense than in the present tense [*β* = −0.930, SE = 0.29, *z* = −3.178, OR = 0.39, *p* < 0.01] and the future tense [*β* = −0.668, SE = 0.28, *z* = −2.356, OR = 0.51, *p* < 0.05]. There was no significant difference between the present and the future tenses [*β* = −0.261, SE = 0.21, *z* = −1.272, OR = 0.77, *p* = 0.41]. The significant effect of aphasia fluency showed that the probability of answering correctly is lower for the speakers with non-fluent aphasia than for the speakers with fluent aphasia [*β* = −1.1367, SE = 0.37, *z* = −3.049, OR = 0.32, *p* < 0.01].

Visual inspections of funnel plots did not reveal any clear asymmetry (see Supplementary materials S6), suggesting the absence of a publication bias.

#### 3.2.2 Objective 2. Effect of task and sociodemographic variables

Given the similar pattern of verbal inflection performance between participants with fluent and non-fluent aphasia, the two groups of participants were grouped in the following analyses. The IPD meta-analyses revealed heterogeneity insofar as the models with all the sources of variability (intercepts for subjects and studies, by subject and by studies random slopes for the effect of tense) fitted the data significantly better than the models without these variability sources (see Supplementary material S4).

##### 3.2.2.1 Task

**Figure 4:**
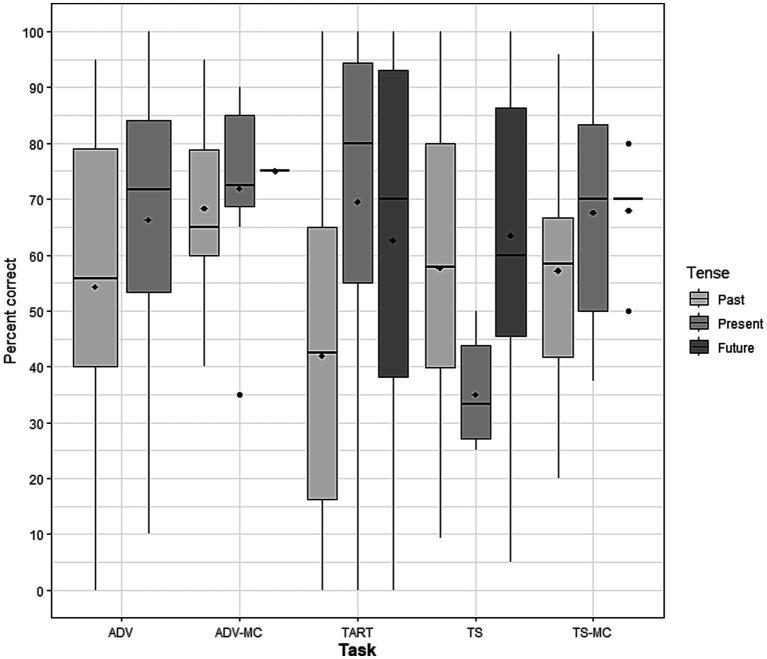
Percent correct as a function of task and tense. Diamonds represent the mean values. TART: Test for Assessing Reference of Time (Bastiaanse et al., 2008); ADV, sentence completion task according to a temporal adverb; ADV-MC, sentence completion task according to a temporal adverb with multiple choice answers; TS, transformational sentence completion task from one time reference to another based on a temporal adverbial; TS-MC, transformational sentence completion task with multiple choice answers.

The model did not find a statistically significant interaction between task and tense [*X*^2^(Martínez-Ferreiro and Bastiaanse, 2013) = 10.79, *p* = 0.15; see Figure 4]. The model showed a significant effect of the task [*X*^2^ (Bastiaanse, 2008) = 20.47, *p* < 0.001]. Post-hoc analyses (with Tukey correction) indicated that the probability of answering correctly was lower in the TART task compared to the Adverb multiple choice task [*β* = −2.1021, SE = 0.56, *z* = −3.732, OR = 0.12, *p* < 0.01] and lower in the adverb task compared to the Adverb multiple choice task [*β* = −1.9908, SE = 0.45, *z* = −4.396, OR = 0.14, *p* < 0.001]. No other differences were significant (see Supplementary materials S5b).

##### 3.2.2.2 Sociodemographic variables

**Figure 5:**
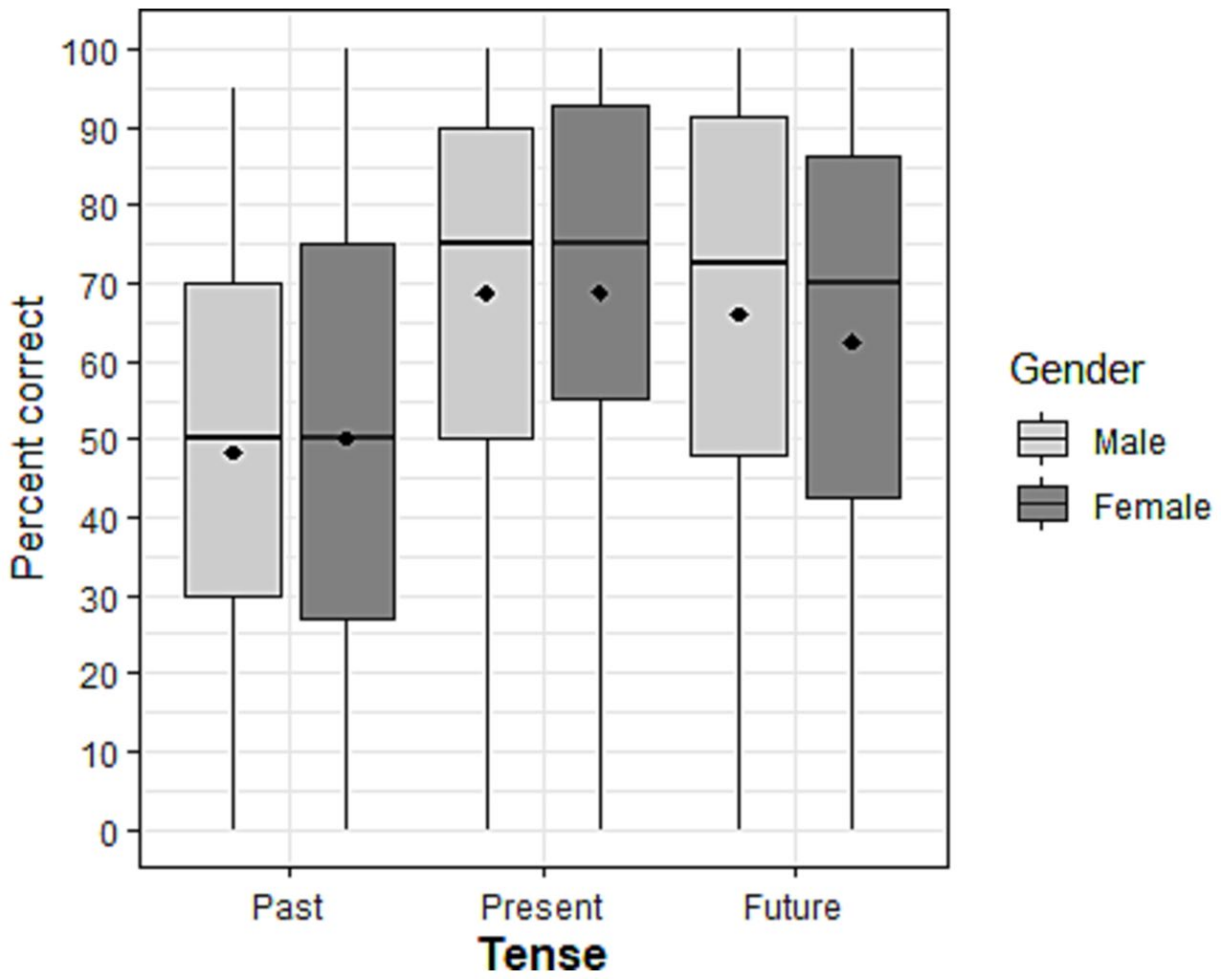
Percent correct as a function of tense and gender. Diamonds represent the mean values.

**Figure 6:**
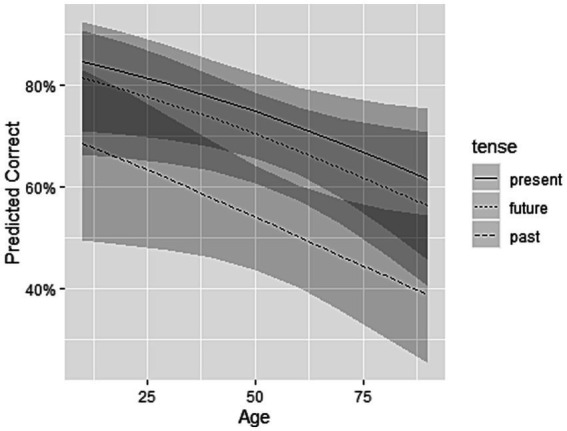
Predicted percent correct as a function of tense and age. The darker shades of gray represent 95% confidence intervals.

The two-way interactions involving tense were non-significant [tense*gender: *X*^2^(2) = 1.427, *p* = 0.49; tense*age: *X*^2^(2) = 4.80, *p* = 0.09], suggesting that the difference between the tenses were similar across gender and ages (see Figure 5, 6). Since the non-significant interactions hid the presence of the main effects, both interactions were thus removed from the final model (see Supplementary material S5c). The final model found no effect of gender [*X*^2^(1) = 0.208, *p* = 0.65], but a significant effect of age [*X*^2^(1) = 4.287, *p* < 0.05], with a lower probability of answering correctly as age increases [*β* = −0.016, SE = 0.008, *z* = −2.105, OR = 0.98, *p* = 0.04].

## 4 Discussion

Our study aimed to characterize tense impairment in people with aphasia. Using a systematic review and an IPD meta-analysis, we analyzed for the first time the differences in the production of tenses (i.e., past, present, future) in people with fluent and non-fluent aphasia and the influence of tasks and socio-demographic variables on time reference.

The systematic review identified 35 studies conducted in 17 languages, with 392 participants. Time reference was assessed by six tasks, mainly the TART, systematically manipulating past tense and one or two other tenses. Twelve studies found dissociations between tenses, mostly to the disadvantage of past tense, and 8 studies found no statistical difference between tenses. Two studies reported mixed results depending on the language assessed or the task used, and 11 studies did not statistically analyze tense differences. The quality of the studies was generally rated as fair or good, except for one study, which was excluded.

An IPD meta-analysis was then carried out on 23 studies for a total of 232 participants (45 with fluent aphasia and 187 with non-fluent aphasia). Our first research question explored whether one tense was more impaired than the others in participants with fluent and non-fluent aphasia. Our analyses showed poorer performance for past tense compared to present and future tenses in both participants with fluent and non-fluent aphasia. These results are congruent with the PADILIH (Bastiaanse et al., 2011), which postulates a selective deficit of past tense in people with aphasia. However, the results must be interpreted with caution for several reasons.

Firstly, most studies included in our meta-analysis (11/23) used the TART, the task developed by the authors of the PADILIH. As discussed in the introduction, this may have resulted in a bias against past tense as this task possibly places a heavier load on working memory in the past condition since the action is not represented in the picture. This working memory load could thus be added to the cognitive load inherent in the past because of the discourse link that has to be established between the narrated event and the moment of enunciation, a link that is unnecessary for the present and future (Zagona, 2003, 2013; Avrutin, 2006). This double load on working memory (task and discourse link) would thus be conducive to poorer performance in past tenses than in non-past tenses. This question of task influence will be discussed in greater depth below.

Secondly, the figures reveal a high degree of inter-individual variability. In a recent study Fyndanis et al. (2018a) found high variability among their participants, with double dissociations in the impaired tenses. These results underline the risk of obtaining biased group analyses, depending on which side the dissociations lean toward. In such cases, individual analyses can be highly instructive. In addition, these findings suggest that there is no single functional origin for verbal inflection difficulties but rather several different origins, depending on the individual’s cognitive and lesion profiles.

Our results also showed that participants with fluent and non-fluent aphasia had a similar performance pattern (i.e., past < present and future), although participants with non-fluent aphasia had poorer performance. These results should be considered with caution given the high inter-individual variability (particularly in participants with fluent aphasia; see Figure 2) and the small number of participants with fluent aphasia compared with those with non-fluent aphasia (45 vs. 187 participants, respectively). More studies, including participants with fluent aphasia, would be necessary to reinforce our conclusions. However, our results align with the few studies directly comparing participants with fluent and non-fluent aphasia on verbal morphology tasks. These studies have generally shown slightly lower accuracy in people with non-fluent aphasia but a similar performance pattern to the one of people with fluent aphasia (Dragoy and Bastiaanse, 2013; Bos and Bastiaanse, 2014; Fyndanis et al., 2018a). In addition, several studies have reported that while performance patterns may be similar between participants with fluent and non-fluent aphasia, the types of errors made may differ. Bos and Bastiaanse (2014) and Kljajevic and Bastiaanse (2010) thus found that participants with fluent aphasia made more “within-target-time-frame errors” (e.g., using the past continuous instead of the simple past), while participants with non-fluent aphasia produced more “outside-target-time-frame errors” (e.g., present tense instead of past tense) and unmarked forms (e.g., infinitives). The authors hypothesized that these patterns would reflect different underlying disorders, with lexical retrieval deficits in fluent aphasia and grammatical encoding deficits in non-fluent aphasia. However, these results are not unanimous. No specific pattern of errors was thus found in participants with fluent aphasia in Jonkers and de Bruin (Jonkers and de Bruin, 2009)'s study, while frame repetition errors were overwhelmingly found in participants with fluent and non-fluent aphasia in Fyndanis et al.’s (2018a) study, which used a transformation task (e.g., yesterday.. ➔ tomorrow..). In our systematic review, we attempted to synthesize the errors made by participants with fluent and non-fluent aphasia reported in 28 studies. However, the wide inter-study variability in terms of participant language, task used, and error categories considered did not allow us to draw a clear picture of error patterns specific to each type of aphasia. More studies analyzing the performance and error patterns of participants with fluent and non-fluent aphasia would strongly contribute to the discussion of similarities and differences in time reference between these two populations.

Our second aim was to analyze the effect of tasks and socio-demographic variables (i.e., age and gender) on time reference. Our results showed a significant task effect, with better performance in the multiple-choice adverb task than in the TART and the verb production task according to a temporal adverb. These results raise the question of the cognitive cost of the tasks used. Indeed, several studies have suggested that tense deficit could manifest only or to a greater extent in tasks that sufficiently challenge the processing system (Faroqi-Shah and Friedman, 2015; Nerantzini et al., 2020; El Ouardi, 2021). Subject-specific factors, such as individuals’ processing resources, may also influence morphosyntactic performance. In the area of syntactic comprehension, several studies have observed different patterns of comprehension performance within the same aphasic syndrome (e.g., Caramazza et al., 2001). The causes of this inter-individual variability are manifold (e.g., limited processing resources or the use of different strategies; Caplan, 2012). In syntactic production, limited working memory capacity has also been shown to affect verbal inflection more than simpler syntactic processes (e.g., agreement) (Fyndanis et al., 2018a). To our knowledge, however, no study in aphasiology has analyzed the links between working memory capacity and performance on various verbal inflection tasks, a gap that needs to be filled in future studies.

Furthermore, only the sentence level was considered in our study. Therefore, it would be interesting to include discursive tasks, such as picture descriptions or interviews, in future studies (Arslan et al., 2016; Siriboonpipattana et al., 2020). Such tasks, which have better ecological validity, could be more cognitively demanding, as they involve message planning, lexical retrieval, and syntactic structure building in addition to verb tense computation. The interaction between these processes has been shown to affect time reference more severely in people with aphasia (Bastiaanse, 2011; Faroqi-Shah and Friedman, 2015). On the other hand, studies in dementia populations have suggested that picture description tasks are more likely to reveal semantic and lexical retrieval deficits than morphosyntactic deficits (Sajjadi et al., 2012; Varlokosta et al., 2023). These findings appear to be supported by a study of participants with aphasia using picture description and interview tasks (Arslan et al., 2016). This study showed that while sentence length and verb diversity were reduced in participants with aphasia, their proportion of inflected verbs was similar to that of healthy participants. Further studies are needed to clarify the time reference profiles of people with aphasia in discourse tasks and to examine whether these profiles are comparable to those observed in sentence-level time reference tasks.

While the cognitive cost of the tasks seems to influence performance in verbal inflection, the respective influence of these tasks on dissociations between tenses remains less clear. In their meta-analysis Faroqi-Shah and Friedman (2015) observed a selective deficit of past and future tenses in only one task, the TART. No significant difference between tenses was observed for the other tasks. In the present study, however, the interaction between tense and task was not significant, suggesting that time reference patterns could be task-independent. One explanation for the discrepancy between these two meta-analyses may lie in the statistical methods used - separate ANOVAs conducted on each task versus mixed-effects regression models. However, future studies comparing tense pattern performance with different tasks would be relevant to shed light on this issue.

Finally, regarding socio-demographic variables, our analyses showed an age effect but no gender effect on time reference. These results seem to fit in with the issue of limited cognitive resources. Indeed, it is now recognized that working memory capacity decreases with age (Klencklen et al., 2017) and impacts morphosyntactic processing (Marini and Andreetta, 2016). As a result, older people with aphasia could be more likely to present difficulties in cognitively-costly verbal inflection tasks. The absence of an age effect in Coulombe et al. (2019) could be explained by the task used, which might not be complex enough to challenge the cognitive resources of the healthy control participants. Concerning gender, no study to our knowledge has investigated its effect on syntactic production. However, in a recent language assessment battery (Joanette et al., 2021), a non-significant effect of gender was found for all subtests, including morphosyntactic comprehension.

### 4.1 Limitations and openings

There are several limitations to our systematic review and IPD meta-analysis. The first one concerns the assessment of study quality. Indeed, no quality assessment tool entirely matched our needs. Consequently, the scale best fitting our study characteristics was retained, but several questions had to be removed. Some older studies were also penalized by the inclusion of a question on the ethical approval given by participants, a piece of information not requested by journals at the time. Despite this, it is worth noting that most studies were judged to be of fair quality, largely due to methodological shortcomings. In future studies, particular attention should be paid to the presentation of the method to encourage replication.

A second limitation concerns the tense categories analyzed. Tenses differ significantly between languages. Some languages have several tenses referring to the present (e.g., English, with the simple present and the present continuous) or to the past (e.g., a perfective and an imperfective form in Greek or French). As these variations are not found in all languages, we had to group them into tense categories. However, this grouping may have masked specific differences linked to these variations. This limitation also raises the general question of differences between languages and their effect on time reference. The participants’ language was not analyzed in our IPD meta-analysis, as it was outside the scope of our review. In addition, the multiplicity of languages (i.e., 14 different languages in the reported studies) and the small number of participants for some languages made it difficult to carry out mixed models. Some studies (Bastiaanse et al., 2011; Bastiaanse, 2013) suggest that the selective deficit in the past may be language-independent. However, more studies should aim to compare several languages or analyze languages that are currently little studied (e.g., French, Hindi, Portuguese) to identify possible cross-language differences.

Thirdly, multilingual participants were removed from our review and IPD meta-analyses. To our knowledge, no study has shown whether plurilingualism influences performance patterns between tenses in aphasia. Without evidence, we preferred to avoid a possible bias linked to plurilingualism by retaining only monolingual participants.

Fourthly, the effect of educational level could not be analyzed because many studies did not report this information. In addition, when it was reported, there was great inter-study variability in the format (i.e., level or years of education) and the way years were counted, as education systems differ between countries. One review (González-Fernández et al., 2011) found an effect of people with aphasia’s education level on several language components after stroke (e.g., oral and written comprehension, oral reading). To our knowledge, no study has investigated the effect of education level on inflection morphology in people with aphasia. However, a recent study with healthy participants (Coulombe et al., 2019) showed no significant correlation between educational level and performance on a verb inflection task. Future studies on time reference should better specify the education level of participants with aphasia so that this effect can be analyzed.

A final limitation relates to bias. Methods for detecting publication bias are poorly-documented in IPD meta-analyses (Ahmed et al., 2012). It would appear, however, that publication bias is reduced in IPD meta-analysis, as the results are derived directly from the raw data and independent of study reports (Stewart et al., 2005). In our meta-analysis, the visual analysis of funnel plots revealed no clear asymmetry between studies. However, we cannot rule out the possibility that potential biases may have influenced our results. One such bias concerns the availability of individual data (Ahmed et al., 2012). In the field of verbal morphology in aphasia, most articles include individual data, which enabled us to include many studies from the outset. We subsequently contacted the authors of the missing articles individually. However, the lack of response from some authors may have impacted the results. Furthermore, we could not find and include unpublished gray literature on the topic despite our research. Finally, other biases, such as those related to the language of publication or outcome reporting, may also have played a role (Sterne et al., 2011).

### 4.2 Conclusion

Our systematic review and IPD meta-analysis are the first to suggest that past tense may be more difficult to produce than other tenses for participants with aphasia, irrespective of the type of aphasia (fluent or non-fluent), albeit with strong inter-individual differences. The tasks used in the studies could differ in complexity, and age could influence the results, with a poorer overall performance in older participants. More studies comparing different task formats in participants with fluent and non-fluent aphasia, and combining qualitative and quantitative group and individual analyses, would significantly improve our understanding of these disorders and their origins. A better understanding of these deficits would then support the development of more targeted treatments, likely to improve the communication skills of people with aphasia.

## Author contributions

NC: Data curation, Formal analysis, Investigation, Writing – original draft; ES: Data curation, Formal analysis, Investigation, Writing – original draft; LZ: Data curation, Writing – review & editing; MF: Conceptualization, Funding acquisition, Project administration, Supervision, Writing – review & editing.
