# Supplementary material for: Time reference in aphasia: are there differences between tenses and aphasia fluency type? A systematic review and individual participant data meta-analysis
Source: Front Psychol. 2024 Feb 8;15:1322539. doi: 10.3389/fpsyg.2024.1322539 (PMC10885357; doi:10.3389/fpsyg.2024.1322539)
Supplement: Supplementary file 1 [file Table_1.DOCX]

Supplementary Material

# S1. R Script

# rm(list=ls())

# library(optimx)

# library(lme4)

# library(sjPlot)

# library(magrittr)

# library(lsmeans)

# library(lattice)

# library(dplyr)

# library(ggplot2)

# # read data

# mydata_long <- read.delim("mydata_long.txt", sep = "\t", stringsAsFactors = TRUE)

# # study_number and case_number as factors

# mydata_long$study_number <- as.factor(mydata_long$study_number)

# mydata_long$case_number <- as.factor(mydata_long$case_number)

# summary(mydata_long)

# ######################################

# # Objective 1: tense and aphasia fluency

# ######################################

# ### Reference = Fluent and present

# # relevel ref = incorr to get the probability of correct and ref = present

# mydata_long$resp <- relevel(mydata_long$resp, ref = "incorr")

# mydata_long$tense <- relevel(mydata_long$tense, ref = "present")

# # Recode variables

# mydata_long$NF<- ifelse(mydata_long$case_aphasia_fluency=="Nonfluent", 1, 0)

# mydata_long$past <- ifelse(mydata_long$tense=="past", 1, 0)

# mydata_long$future <- ifelse(mydata_long$tense=="future", 1, 0)

# # check code

# table(mydata_long$case_aphasia_fluency,mydata_long$NF)

# table(mydata_long$tense,mydata_long$past)

# table(mydata_long$tense,mydata_long$future)

# # create interaction terms

# mydata_long$NF_past = mydata_long$NF * mydata_long$past

# mydata_long$NF_future = mydata_long$NF * mydata_long$future

# summary(mydata_long)

# # model with interaction

# model_glmer_long_axt <- glmer(resp~ NF + (past + future) + (NF_past + NF_future) + (1+tense|study_number/case_number), data = mydata_long, family="binomial", control = glmerControl(optimizer ='bobyqa', optCtrl=list(maxfun=100000)))

# summary(model_glmer_long_axt)

# tab_model(model_glmer_long_axt, transform = NULL)

# ### test heterogeneity

# # model without case_number

# model_glmer_NoCase <- glmer(resp ~ NF + (past + future) + (NF_past + NF_future) + (1+tense|study_number), data = mydata_long, family="binomial", control=glmerControl(optimizer="bobyqa", optCtrl=list(maxfun=100000)))

# summary(model_glmer_NoCase)

# anova(model_glmer_long_axt, model_glmer_NoCase)

# # model without study_number

# model_glmer_NoStudy <- glmer(resp ~ NF + (past + future) + (NF_past + NF_future) + (1+tense|case_number), data = mydata_long, family="binomial", control=glmerControl(optimizer="bobyqa", optCtrl=list(maxfun=100000)))

# summary(model_glmer_NoStudy)

# anova(model_glmer_long_axt, model_glmer_NoStudy)

# # Model without random slope

# model_glmer_long_axt2 <- glmer(resp ~ NF + (past + future) + (NF_past + NF_future) + (1|study_number/case_number), data = mydata_long, family="binomial", control = glmerControl(optimizer ='bobyqa', optCtrl=list(maxfun=100000)))

# summary(model_glmer_long_axt2)

# anova(model_glmer_long_axt, model_glmer_long_axt2)

# ### Test interaction and main effects

# # model without interaction

# model_glmer_long_at <- glmer(resp~ NF + (past + future) + (1+tense|study_number/case_number), data = mydata_long, family="binomial", control = glmerControl(optimizer ='bobyqa', optCtrl=list(maxfun=100000)))

# summary(model_glmer_long_at)

# anova(model_glmer_long_axt, model_glmer_long_at )

# # Model without aphasia fluency

# model_glmer_long_a <- glmer(resp~ (past + future) + (NF_past + NF_future) + (1+tense|study_number/case_number), data = mydata_long, family="binomial", control = glmerControl(optimizer ='bobyqa', optCtrl=list(maxfun=100000)))

# summary(model_glmer_long_a)

# anova(model_glmer_long_axt, model_glmer_long_a)

# # Model without tense

# model_glmer_long_t <- glmer(resp~NF + (NF_past + NF_future) + (1+tense|study_number/case_number), data = mydata_long, family="binomial", control = glmerControl(optimizer ='bobyqa', optCtrl=list(maxfun=100000)))

# summary(model_glmer_long_t)

# anova(model_glmer_long_axt, model_glmer_long_t)

# # Comparison 2 by 2 for tense

# model_tense <- glmer(resp ~ tense + (1+tense|study_number/case_number), family = binomial, data = mydata_long, control=glmerControl(optimizer = "bobyqa", optCtrl = list(maxfun = 1e+05)))

# tense_lsm <- emmeans(model_tense, ~ tense , cov.reduce = FALSE)

# tense_pairs<-pairs(tense_lsm)

# tense_pairs

# ### Analyze of the residues

# ### global normality

# mydata_long$residuals <- resid(model_glmer_long_axt)

# hist(mydata_long$residuals)

# qqnorm(mydata_long$residuals)

# qqline(mydata_long$residuals)

# ### fitted residues

# plot(model_glmer_long_axt)

# # normality Participant

# qqnorm(ranef(model_glmer_long_axt)$case_number[,1])

# qqline(ranef(model_glmer_long_axt)$case_number[,1])

# ranefModel <- ranef(model_glmer_long_axt, condVar = TRUE)

# dotplot(ranefModel)

# library(DHARMa)

# resid_model_glmer_long_axt <- simulateResiduals(fittedModel = model_glmer_long_axt)

# #############################################################

# # Objective 2: effect of sociodemographic and task variables

# #############################################################

# ########

# ### Tasks

# ########

# # reference = TART

# mydata_long$task <- relevel(mydata_long$task, ref = "TART")

# ## recode tense

# mydata_long$past <- ifelse(mydata_long$tense=="past", 1, 0)

# mydata_long$future <- ifelse(mydata_long$tense=="future", 1, 0)

# # recode task

# mydata_long$Adverb <- ifelse(mydata_long$task=="Adverb", 1, 0)

# mydata_long$Adverb_CM <- ifelse(mydata_long$task=="Adverb_CM", 1, 0)

# mydata_long$Transf_source <- ifelse(mydata_long$task=="Transf_source", 1, 0)

# mydata_long$Transf_source_CM <- ifelse(mydata_long$task=="Transf_source_CM", 1, 0)

# #check code

# table(mydata_long$task,mydata_long$Adverb)

# table(mydata_long$task,mydata_long$Adverb_CM)

# table(mydata_long$task,mydata_long$Transf_source)

# table(mydata_long$task,mydata_long$Transf_source_CM)

# #create interaction terms

# mydata_long$past_Adverb = mydata_long$past * mydata_long$Adverb

# mydata_long$past_Adverb_CM = mydata_long$past * mydata_long$Adverb_CM

# mydata_long$past_Transf_source = mydata_long$past * mydata_long$Transf_source

# mydata_long$past_Transf_source_CM = mydata_long$past * mydata_long$Transf_source_CM

# mydata_long$future_Adverb = mydata_long$future * mydata_long$Adverb

# mydata_long$future_Adverb_CM = mydata_long$future * mydata_long$Adverb_CM

# mydata_long$future_Transf_source = mydata_long$future * mydata_long$Transf_source

# mydata_long$future_Transf_source_CM = mydata_long$future * mydata_long$Transf_source_CM

# # model with interaction tense x task

# model_glmer_long_txt <- glmer(resp~past + future + Adverb + Adverb_CM + Transf_source+ Transf_source_CM + past_Adverb + past_Adverb_CM + past_Transf_source + past_Transf_source_CM + future_Adverb+future_Adverb_CM + future_Transf_source + future_Transf_source_CM + (1+tense|study_number/case_number), data = mydata_long, family="binomial", control = glmerControl(optimizer ='bobyqa', optCtrl=list(maxfun=100000)))

# summary(model_glmer_long_txt)

# tab_model(model_glmer_long_txt, transform = NULL)

# ### test heterogeneity

# # model without case_number

# model_glmer_NoCase_task <- glmer(resp~past + future + Adverb + Adverb_CM + Transf_source+ Transf_source_CM + past_Adverb + past_Adverb_CM + past_Transf_source + past_Transf_source_CM + future_Adverb + future_Adverb_CM + future_Transf_source + future_Transf_source_CM + (1+tense|study_number), data = mydata_long, family="binomial", control=glmerControl(optimizer="bobyqa", optCtrl=list(maxfun=100000)))

# summary(model_glmer_NoCase_task)

# anova(model_glmer_long_txt, model_glmer_NoCase_task)

# # model without study_number

# model_glmer_NoStudy_task <- glmer(resp~past + future + Adverb + Adverb_CM + Transf_source+ Transf_source_CM + past_Adverb + past_Adverb_CM + past_Transf_source + past_Transf_source_CM + future_Adverb + future_Adverb_CM + future_Transf_source + future_Transf_source_CM + (1+tense|case_number), data = mydata_long, family="binomial", control=glmerControl(optimizer="bobyqa", optCtrl=list(maxfun=100000)))

# summary(model_glmer_NoStudy)

# anova(model_glmer_long_txt, model_glmer_NoStudy_task)

# # Model without random slope

# model_glmer_long_txt2 <- glmer(resp~past + future + Adverb + Adverb_CM + Transf_source+ Transf_source_CM + past_Adverb + past_Adverb_CM + past_Transf_source + past_Transf_source_CM + future_Adverb + future_Adverb_CM + future_Transf_source + future_Transf_source_CM + (1|study_number/case_number), data = mydata_long, family="binomial", control = glmerControl(optimizer ='bobyqa', optCtrl=list(maxfun=100000)))

# summary(model_glmer_long_axt2)

# anova(model_glmer_long_txt, model_glmer_long_txt2)

# ### Test interaction and main effects

# # model without interaction

# model_glmer_long_tt <- glmer(resp~past + future + Adverb + Adverb_CM + Transf_source+ Transf_source_CM + (1+tense|study_number/case_number), data = mydata_long, family="binomial", control = glmerControl(optimizer ='bobyqa', optCtrl=list(maxfun=100000)))

# summary(model_glmer_long_tt)

# anova(model_glmer_long_txt, model_glmer_long_tt )

# # Model without task

# model_glmer_long_task <- glmer(resp~past + future + past_Adverb+past_Adverb_CM+past_Transf_source + past_Transf_source_CM + future_Adverb+future_Adverb_CM + future_Transf_source + future_Transf_source_CM + (1+tense|study_number/case_number), data = mydata_long, family="binomial", control = glmerControl(optimizer ='bobyqa', optCtrl=list(maxfun=100000)))

# summary(model_glmer_long_task)

# anova(model_glmer_long_txt, model_glmer_long_task)

# # Comparison 2 by 2 for task

# model_task <- glmer(resp ~ task + (1+tense|study_number/case_number), family = binomial, data = mydata_long, control=glmerControl(optimizer = "bobyqa", optCtrl = list(maxfun = 1e+05)))

# task_lsm <- emmeans(model_task, ~ task , cov.reduce = FALSE)

# task_pairs<-pairs(task_lsm)

# task_pairs

# ### Analyze of the residues

# ### global normality

# mydata_long$residuals <- resid(model_glmer_long_txt)

# hist(mydata_long$residuals)

# qqnorm(mydata_long$residuals)

# qqline(mydata_long$residuals)

# ### fitted residues

# plot(model_glmer_long_txt)

# # normality Participant

# qqnorm(ranef(model_glmer_long_txt)$case_number[,1])

# qqline(ranef(model_glmer_long_txt)$case_number[,1])

# ranefModel <- ranef(model_glmer_long_txt, condVar = TRUE)

# dotplot(ranefModel)

# library(DHARMa)

# resid_model_glmer_long_txt <- simulateResiduals(fittedModel = model_glmer_long_txt)

# plot(resid_model_glmer_long_txt)

# ##########################

# ### sociodemographic variables

# ##########################

# mydata_long$miss_age_gender <- is.na(mydata_long$case_age) & is.na(mydata_long$case_gender)

# ## recode tense

# mydata_long$past <- ifelse(mydata_long$tense=="past", 1, 0)

# mydata_long$future <- ifelse(mydata_long$tense=="future", 1, 0)

# # recode gender

# mydata_long$M <- ifelse(mydata_long$case_gender=="M", 1, 0)

# #check code

# table(mydata_long$tense,mydata_long$past)

# table(mydata_long$tense,mydata_long$future)

# table(mydata_long$case_gender,mydata_long$M)

# #create interaction terms

# mydata_long$age_past = mydata_long$case_age * mydata_long$past

# mydata_long$age_future = mydata_long$case_age * mydata_long$future

# mydata_long$M_past = mydata_long$M * mydata_long$past

# mydata_long$M_future = mydata_long$M * mydata_long$future

# summary(mydata_long)

# ## model with interaction

# model_glmer_long_txgxage <- glmer(resp~ (past + future + M + case_age + age_past + age_future+ M_past+ M_future) + (1+tense|study_number/case_number), data = mydata_long[mydata_long$miss_age_gender==FALSE,], family="binomial", control = glmerControl(optimizer ='bobyqa', optCtrl=list(maxfun=100000)))

# summary(model_glmer_long_txgxage)

# #### test heterogeneity

# # model without case_number

# model_glmer_NoCase_genderage <- glmer(resp~ (past + future + M + case_age + age_past + age_future+ M_past+ M_future)

# + (1+tense|study_number), data = mydata_long[mydata_long$miss_age_gender==FALSE,], family="binomial", control=glmerControl(optimizer="bobyqa", optCtrl=list(maxfun=100000)))

# summary(model_glmer_NoCase_genderage)

# anova(model_glmer_long_txgxage, model_glmer_NoCase_genderage)

# # model without study_number

# model_glmer_NoStudy_genderage <- glmer(resp~ (past + future + M + case_age + age_past + age_future+ M_past+ M_future)

# + (1+tense|case_number), data = mydata_long[mydata_long$miss_age_gender==FALSE,], family="binomial", control=glmerControl(optimizer="bobyqa", optCtrl=list(maxfun=100000)))

# summary(model_glmer_NoStudy_genderage)

# anova(model_glmer_long_txgxage, model_glmer_NoStudy_genderage)

# # Model without random slope

# model_glmer_long_txgxage2 <- glmer(resp~ (past + future + M + case_age + age_past + age_future+ M_past+ M_future)

# + (1|study_number/case_number), data = mydata_long[mydata_long$miss_age_gender==FALSE,], family="binomial", control = glmerControl(optimizer ='bobyqa', optCtrl=list(maxfun=100000)))

# summary(model_glmer_long_txgxage2)

# anova(model_glmer_long_txgxage, model_glmer_long_txgxage2)

# ### Test interaction and main effects

# # model without interaction age and without interaction gender

# model_glmer_long_tgage <- glmer(resp~ (past + future + M + case_age) + (1+tense|study_number/case_number), data = mydata_long[mydata_long$miss_age_gender==FALSE,], family="binomial", control = glmerControl(optimizer ='bobyqa', optCtrl=list(maxfun=100000)))

# summary(model_glmer_long_tgage)

# tab_model(model_glmer_long_tgage, transform = NULL)

# # model without interaction tense*age

# model_glmer_long_txgage <- glmer(resp~ (past + future + M + case_age + M_past+ M_future) + (1+tense|study_number/case_number), data = mydata_long[mydata_long$miss_age_gender==FALSE,], family="binomial", control = glmerControl(optimizer ='bobyqa', optCtrl=list(maxfun=100000)))

# summary(model_glmer_long_txgage)

# anova(model_glmer_long_txgxage, model_glmer_long_txgage)

# # model without interaction tense*gender

# model_glmer_long_tgxage <- glmer(resp~ (past + future + M + case_age + age_past + age_future) + (1+tense|study_number/case_number), data = mydata_long[mydata_long$miss_age_gender==FALSE,], family="binomial", control = glmerControl(optimizer ='bobyqa', optCtrl=list(maxfun=100000)))

# summary(model_glmer_long_tgxage)

# anova(model_glmer_long_txgxage, model_glmer_long_tgxage)

# # Since the non-significant interactions hid the presence of the main effects, they were removed from the final model.

# # Final model

# model_glmer_long_tgage <- glmer(resp~ (past + future + M + case_age) + (1+tense|study_number/case_number), data = mydata_long[mydata_long$miss_age_gender==FALSE,], family="binomial", control = glmerControl(optimizer ='bobyqa', optCtrl=list(maxfun=100000)))

# summary(model_glmer_long_tgage)

# # Model without age

# model_glmer_long_age <- glmer(resp~ (past + future + M) + (1+tense|study_number/case_number), data = mydata_long[mydata_long$miss_age_gender==FALSE,], family="binomial", control = glmerControl(optimizer ='bobyqa', optCtrl=list(maxfun=100000)))

# summary(model_glmer_long_age)

# anova(model_glmer_long_tgage, model_glmer_long_age)

# # Model without gender

# model_glmer_long_gender <- glmer(resp~ (past + future + case_age) + (1+tense|study_number/case_number), data = mydata_long[mydata_long$miss_age_gender==FALSE,], family="binomial", control = glmerControl(optimizer ='bobyqa', optCtrl=list(maxfun=100000)))

# summary(model_glmer_long_gender)

# anova(model_glmer_long_tgage, model_glmer_long_gender)

# ### Analyze of the residues

# ### global normality

# mydata_long[mydata_long$miss_age_gender==FALSE,]$residuals <- resid(model_glmer_long_tgage)

# hist(mydata_long[mydata_long$miss_age_gender==FALSE,]$residuals)

# qqnorm(mydata_long[mydata_long$miss_age_gender==FALSE,]$residuals)

# qqline(mydata_long[mydata_long$miss_age_gender==FALSE,]$residuals)

# ### fitted residues

# plot(model_glmer_long_tgage)

# # normality Participant

# qqnorm(ranef(model_glmer_long_tgage)$case_number[,1])

# qqline(ranef(model_glmer_long_tgage)$case_number[,1])

# ranefModel <- ranef(model_glmer_long_tgage, condVar = TRUE)

# dotplot(ranefModel)

# library(DHARMa)

# resid_model_glmer_long_tgage <- simulateResiduals(fittedModel = model_glmer_long_tgage)

# plot(resid_model_glmer_long_tgage)

# ######################

# ### R Version for analyses

# ######################

# sessionInfo()

# R version 4.3.1 (2023-06-16)

# Platform: aarch64-apple-darwin22.4.0 (64-bit)

# Running under: macOS Ventura 13.5

# S2. Excel sheet with individual data

See additional indexed material

# S3. Experimental tasks used in the studies: examples

| Task | Example |
| --- | --- |
| Sentence completion task according to a temporal adverb | This is the man who now ……the garden (water) |
| Sentence completion task according to a temporal adverb with multiple choice answers | Tomorrow the man …… the garden  (response options: will water, was watering, watered). |
| Test for Assessing Reference of Time (Bastiaanse, Jonkers, & Thompson, 2008) | This is the man who has just planted the flower.  This is the man who ……  (answer: has just watered the garden) |
| Transformational sentence completion task | Tomorrow, the gardener will water the garden.  Yesterday, the gardener ……  (target: watered the garden) |
| Transformational sentence completion task with multiple choice answers | Tomorrow, he will water.  Yesterday, he ……  (response options: will water, watered). |

# S4. Heterogeneity analyses

To test heterogeneity, likelihood ratio tests were used to compare the models with the sources of variance (intercepts for subjects and studies, by subject and by studies random slopes for the effect of tense) to models without them.

Table S4a. Model Tense x aphasia fluency (objective 1)

| Glmer Model | | |
| --- | --- | --- |
| glmer(resp~ NF + (past + future) + (NF_past + NF_future) + 1+tense\|study_number/case_number), data = mydata_long, family="binomial", control = glmerControl(optimizer ='bobyqa', optCtrl=list(maxfun=100000))) | | |
| Random effect removed | Likelihood ratio test |  |
| Participants | X^2^(6) = 2904.4, *p* < .001 |  |
| Studies | X^2^(6) = 77.15, *p* < .001 |  |
| Random slope for the effect of tense | X^2^(10) = 1176.5, *p* < .001 |  |

Table S4b. Model Tense x Task (objective 2a)

| Glmer Model | | |
| --- | --- | --- |
| glmer(resp~past + future + Adverb + Adverb_CM + Transf_source + Transf_source_CM + past_Adverb+past_Adverb_CM + past_Transf_source + past_Transf_source_CM + future_Adverb + future_Adverb_CM + future_Transf_source + future_Transf_source_CM + (1+tense\|study_number/case_number), data = mydata_long, family="binomial", control = glmerControl(optimizer ='bobyqa', optCtrl=list(maxfun=100000))) | | |
| Random effect removed | Likelihood ratio test |  |
| Participants | X^2^(6) = 3009.9, *p* < .001 |  |
| Studies | X^2^(6) = 67.38, *p* < .001 |  |
| Random slope for the effect of tense | X^2^(10) = 946.34, *p* < .001 |  |

Table S4c. Model Tense x Gender X Age (Objective 2b)

| Glmer Model | | |
| --- | --- | --- |
| glmer(resp~ (past + future + M + case_age + age_past + age_future+ M_past+ M_future) + (1+tense\|study_number/case_number), data = mydata_long[mydata_long$miss_age_gender==FALSE,], family="binomial", control = glmerControl(optimizer ='bobyqa', optCtrl=list(maxfun=100000))) | | |
| Random effect removed | Likelihood ratio test |  |
| Participants | X^2^(6) = 2683, *p* < .001 |  |
| Studies | X^2^(6) = 88.26, *p* < .001 |  |
| Random slope for the effect of tense | X^2^(10) = 1202, *p* < .001 |  |

# S5. Results of the mixed-effects models

Table S5a. Summary of the mixed-effects logistic regression models for the tense and aphasia fluency

| **Predictors** | **Log-Odds** | **CI** | **p** | **Likelihood ratio tests** |
| --- | --- | --- | --- | --- |
| (Intercept) | 1.84 | 1.08 – 2.60 | **<0.001** |  |
| **tense** |  |  |  | χ^2^(2) = 7.07, p = .029 |
| tense [past] | -1.11 | -1.93 – -0.29 | **0.008** |  |
| tense [future] | -0.93 | -1.90 – 0.04 | 0.060 |  |
| **aphasia** |  |  |  | χ^2^(1) = 9.07, p = .003 |
| case aphasia fluency [Nonfluent] | -1.14 | -1.87 – -0.41 | **0.002** |  |
| **tense*aphasia** |  |  |  | χ^2^(2) = 2.28, p = .321 |
| tense [past] * case aphasia fluency [Nonfluent] | 0.20 | -0.50 – 0.89 | 0.580 |  |
| tense [future] * case aphasia fluency [Nonfluent] | 0.76 | -0.22 – 1.74 | 0.129 |  |

*Note.* The reference levels are present for “tense” and fluent for “aphasia”

S5b. Summary of the mixed-effects logistic regression models for the tense and task

| **Predictors** | **Log-Odds** | **CI** | **p** | **Likelihood ratio tests** |
| --- | --- | --- | --- | --- |
| (Intercept) | 1.07 | 0.44 – 1.71 | **0.001** |  |
| **tense** |  |  |  | χ2(2) = 13.88, p < .001 |
| tense [past] | -1.62 | -2.33 – -0.91 | **<0.001** |  |
| tense [future] | -0.42 | -0.86 – 0.03 | 0.068 |  |
| **task** |  |  |  | χ2(4) = 20.47, p < .001 |
| task [Adverb] | -0.78 | -1.98 – 0.43 | 0.207 |  |
| task [Adverb_CM] | 1.73 | 0.21 – 3.24 | **0.026** |  |
| task [Transf_source] | -1.25 | -2.64 – 0.14 | 0.077 |  |
| task [Transf_source_CM] | -0.04 | -1.71 – 1.62 | 0.961 |  |
| **interaction** |  |  |  | χ2(7) = 10.79, p = .148 |
| tense [past] * task [Adverb] | 1.32 | -0.02 – 2.67 | 0.054 |  |
| tense [past] * task [Adverb_CM] | 0.35 | -1.40 – 2.10 | 0.694 |  |
| tense [past] * task [Transf_source] | 2.26 | 0.79 – 3.72 | **0.003** |  |
| tense [past] * task [Transf_source_CM] | 0.95 | -0.85 – 2.76 | 0.301 |  |
| tense [future] * task [Adverb_CM] | -0.38 | -3.40 – 2.65 | 0.807 |  |
| tense [future] * task [Transf_source] | 1.20 | -0.01 – 2.41 | 0.052 |  |
| tense [future] * task [Transf_source_CM] | 0.14 | -1.54 – 1.83 | 0.867 |  |

*Note.* The reference levels are present for “tense” and TART for “task”

S5c. Summary of the mixed-effects logistic regression models for the tense, gender and age (without the interaction)

| *Predictors* | *Log-Odds* | *CI* | *p* |
| --- | --- | --- | --- |
| (Intercept) | 1.84 | 0.89 – 2.79 | **<0.001** |
| tense [past] | -0.92 | -1.53 – -0.32 | **0.003** |
| tense [future] | -0.22 | -0.65 – 0.22 | 0.336 |
| case gender [M] | 0.10 | -0.32 – 0.51 | 0.647 |
| case age | -0.02 | -0.03 – -0.00 | **0.035** |

*Note.* The reference levels are present for “tense” and Female for “Gender”

# S6. Funnel plots


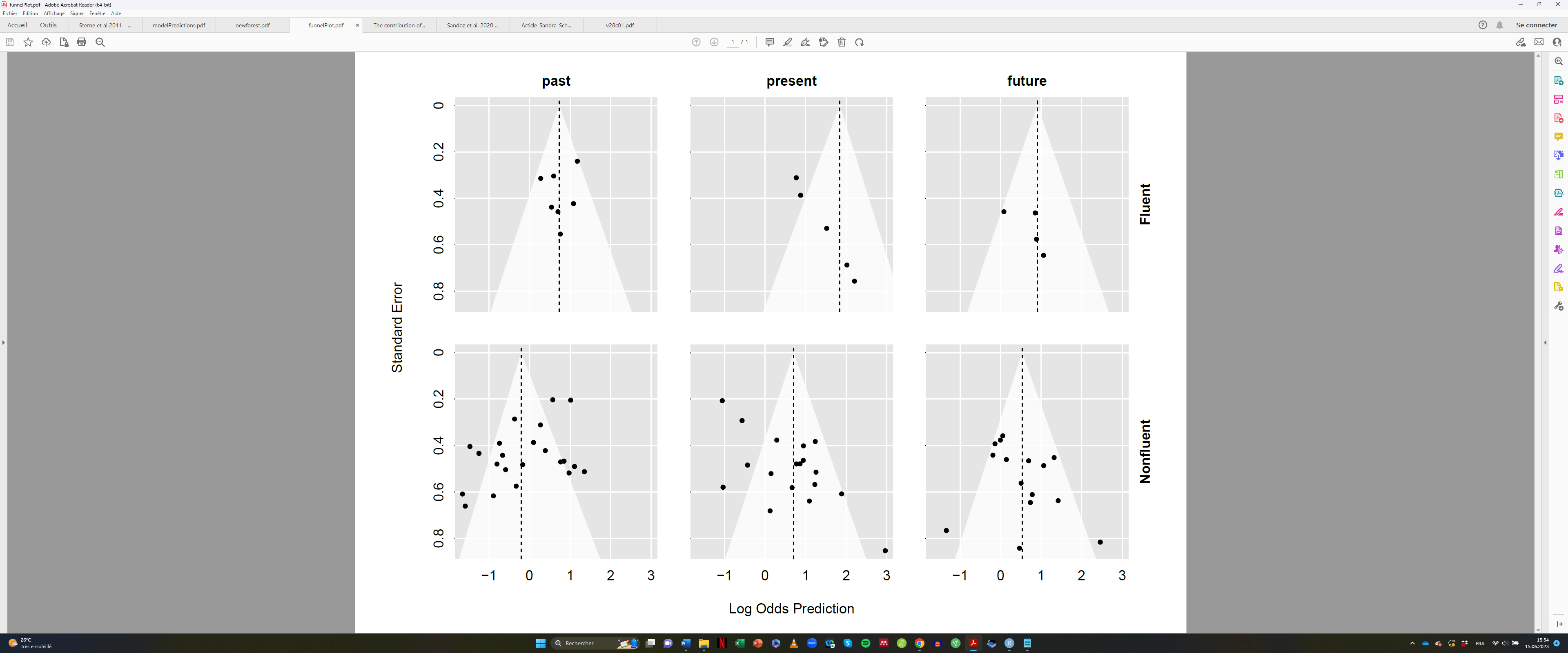


Funnel plots of the effect size estimates (log odd) for the participants with fluent and non-fluent aphasia in each tense.

The funnel plots depict the uncertainty of model estimates of a given study, expressed as the standard error of the study model estimates (on a reversed scale), as a function of the strength of effect in the study, expressed as the deviation from the (fixed effects) model prediction across all studies, for the three tenses and two fluency levels. Hence, each point represents an individual study (for the given tense and fluency combination). The shaded area represents the 95% confidence region, hence the area within which 95% of the points should lie in the absence of publication bias.


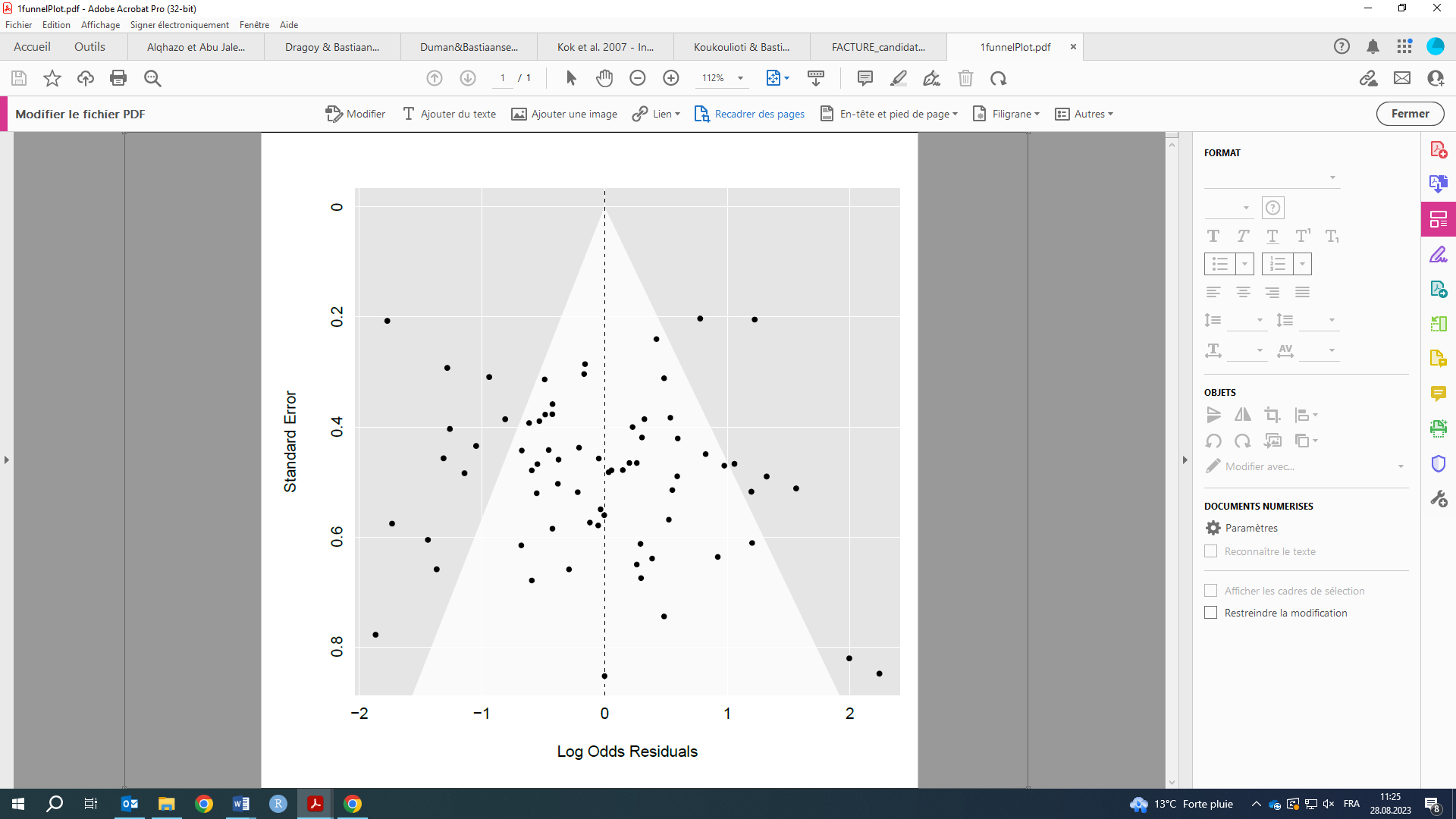


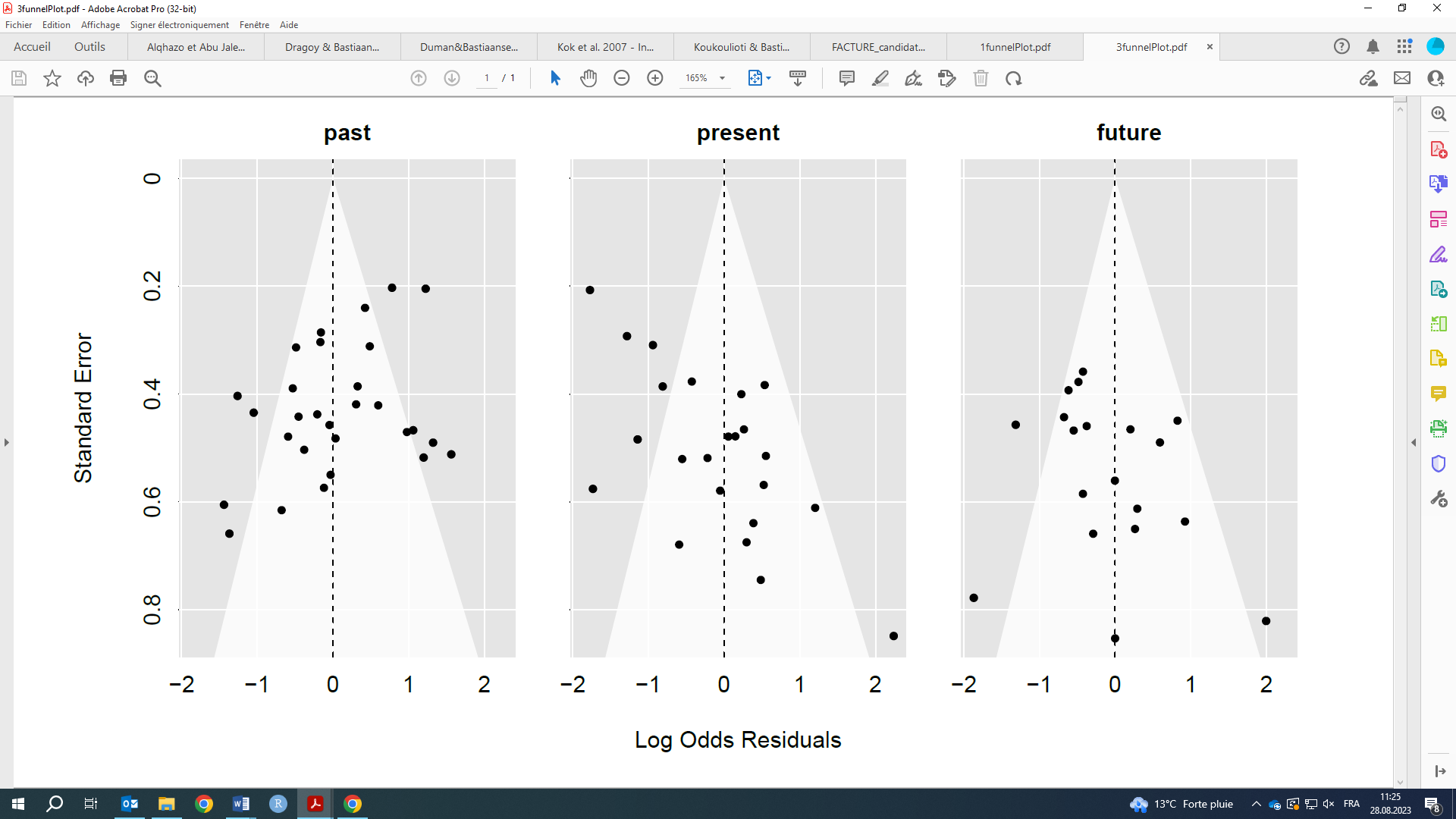


Funnel plots of the effect size estimates (log odd) for all the participants with aphasia in all tenses and in each tense.
